# Supplementary material for: Aggregation-resistant alpha-synuclein tetramers are reduced in the blood of Parkinson’s patients
Source: EMBO Mol Med. 2024 Jun 5;16(7):10. doi: 10.1038/s44321-024-00083-5 (PMC11250827; doi:10.1038/s44321-024-00083-5)
Supplement: Supplementary file 1 — Table EV1 [file 44321_2024_83_MOESM1_ESM.docx]

Table EV1 Summary list of human proteins identified via mass spectrometry in blood-derived purified 60 kDa α-synuclein (IP α-synuclein 211) compared to background (mock IP samples)

| **Sample** | **Accession number** | **Description of protein identified in the sample** | **Species** |
| --- | --- | --- | --- |
| α-synuclein 211 only (α-synuclein) IP | P37840 | Alpha-synuclein | Homo Sapiens |
| α-synuclein 211 + mock IP | P01625 | Ig kappa-V-IV chain | Homo Sapiens |
| α-synuclein 211 + mock IP | P68871 | Hemoglobin subunit beta | Homo Sapiens |
| α-synuclein 211 + mock IP | P69905 | Hemoglobin subunit alpha | Homo Sapiens |

The table shows the results for the mock samples and the α-synuclein results after background substraction using the mock samples. α-Synuclein is the only protein specifically associated with the 60 kDa band characterized as α-synuclein tetramer.
