# Supplementary material for: Aggregation-resistant alpha-synuclein tetramers are reduced in the blood of Parkinson’s patients
Source: EMBO Mol Med. 2024 Jun 5;16(7):10. doi: 10.1038/s44321-024-00083-5 (PMC11250827; doi:10.1038/s44321-024-00083-5)
Supplement: Supplementary file 3 — Source data Fig. 2 [file 44321_2024_83_MOESM3_ESM.zip › Source Data Fig. 2/Fig. 2_Blot No. and signal intensities.docx]

Summary of raw signal intensities from the LI-COR Image Studio Software for Fig. 2

| Image Name | Channel | Cohort | Classification | Subject | Crosslinker | Crosslinker f.c. | Technical replicate | kDa band | Signal intensity |
| --- | --- | --- | --- | --- | --- | --- | --- | --- | --- |
| 0000747_01 | 800 | UK | Control | #1 | DSG | 0.25 | 1 | 60 | 8701,154541 |
| 0000747_01 | 800 | UK | Control | #1 | DSG | 0.25 | 1 | 14 | 857,2192383 |
| 0000747_01 | 800 | UK | Control | #1 | DSG | 0.25 | 2 | 60 | 8485,972534 |
| 0000747_01 | 800 | UK | Control | #1 | DSG | 0.25 | 2 | 14 | 773,4199219 |
| 0000747_01 | 800 | UK | Control | #1 | DSG | 1.43 | 1 | 60 | 12371,66113 |
| 0000747_01 | 800 | UK | Control | #1 | DSG | 1.43 | 1 | 14 | 199,6738281 |
| 0000747_01 | 800 | UK | Control | #1 | DSG | 1.43 | 2 | 60 | 13709,43237 |
| 0000747_01 | 800 | UK | Control | #1 | DSG | 1.43 | 2 | 14 | 319,6826172 |
| 0000752_01 | 800 | UK | Control | #1 | GA | 0.0067 | 1 | 60 | 6852,038574 |
| 0000752_01 | 800 | UK | Control | #1 | GA | 0.0067 | 1 | 14 | 1023,226563 |
| 0000752_01 | 800 | UK | Control | #1 | GA | 0.0067 | 2 | 60 | 6293,95459 |
| 0000752_01 | 800 | UK | Control | #1 | GA | 0.0067 | 2 | 14 | 875,7226563 |
| 0000752_01 | 800 | UK | Control | #1 |  | 0 |  | 60 | 1048,492188 |
| 0000752_01 | 800 | UK | Control | #1 |  | 0 |  | 14 | 3549,830078 |
| 0000747_01 | 800 | UK | Control | #1 |  | 0 |  | 60 | 1368,698975 |
| 0000747_01 | 800 | UK | Control | #1 |  | 0 |  | 14 | 1090,135254 |
| 0000747_01 | 800 | UK | Control | #2 | DSG | 0.25 | 1 | 60 | 11966,00684 |
| 0000747_01 | 800 | UK | Control | #2 | DSG | 0.25 | 1 | 14 | 1651,988281 |
| 0000747_01 | 800 | UK | Control | #2 | DSG | 0.25 | 2 | 60 | 6161,565918 |
| 0000747_01 | 800 | UK | Control | #2 | DSG | 0.25 | 2 | 14 | 1076,579102 |
| 0000747_01 | 800 | UK | Control | #2 | DSG | 1.43 | 1 | 60 | 13959,47534 |
| 0000747_01 | 800 | UK | Control | #2 | DSG | 1.43 | 1 | 14 | 242,2104492 |
| 0000747_01 | 800 | UK | Control | #2 | DSG | 1.43 | 2 | 60 | 13832,07764 |
| 0000747_01 | 800 | UK | Control | #2 | DSG | 1.43 | 2 | 14 | 295,4526367 |
| 0000752_01 | 800 | UK | Control | #2 | GA | 0.0067 | 1 | 60 | 5611,115234 |
| 0000752_01 | 800 | UK | Control | #2 | GA | 0.0067 | 1 | 14 | 332,3129883 |
| 0000752_01 | 800 | UK | Control | #2 | GA | 0.0067 | 2 | 60 | 5652,358887 |
| 0000752_01 | 800 | UK | Control | #2 | GA | 0.0067 | 2 | 14 | 367,0092773 |
| 0000752_01 | 800 | UK | Control | #2 |  | 0 |  | 60 | 935,5136719 |
| 0000752_01 | 800 | UK | Control | #2 |  | 0 |  | 14 | 1773,315918 |
| 0000747_01 | 800 | UK | Control | #2 |  | 0 |  | 60 | 2433,505615 |
| 0000747_01 | 800 | UK | Control | #2 |  | 0 |  | 14 | 1307,456543 |
| 0000752_01 | 800 | UK | Control | #3 | DSG | 0.25 | 1 | 60 | 6857,396484 |
| 0000752_01 | 800 | UK | Control | #3 | DSG | 0.25 | 1 | 14 | 3758,353027 |
| 0000752_01 | 800 | UK | Control | #3 | DSG | 0.25 | 2 | 60 | 6378,61084 |
| 0000752_01 | 800 | UK | Control | #3 | DSG | 0.25 | 2 | 14 | 2775,396973 |
| 0001131_01 | 800 | UK | Control | #3 | DSG | 1.43 | 1 | 60 | 1036,191406 |
| 0001131_01 | 800 | UK | Control | #3 | DSG | 1.43 | 1 | 14 | 1965,13916 |
| 0001131_01 | 800 | UK | Control | #3 | DSG | 1.43 | 2 | 60 | 1123,451172 |
| 0001131_01 | 800 | UK | Control | #3 | DSG | 1.43 | 2 | 14 | 1786,474609 |
| 0000752_01 | 800 | UK | Control | #3 | GA | 0.0067 | 1 | 60 | 7318,191895 |
| 0000752_01 | 800 | UK | Control | #3 | GA | 0.0067 | 1 | 14 | 1263,956299 |
| 0000752_01 | 800 | UK | Control | #3 | GA | 0.0067 | 2 | 60 | 7340,987793 |
| 0000752_01 | 800 | UK | Control | #3 | GA | 0.0067 | 2 | 14 | 1184,233398 |
| 0000752_01 | 800 | UK | Control | #3 |  | 0 |  | 60 | 1565,331787 |
| 0000752_01 | 800 | UK | Control | #3 |  | 0 |  | 14 | 4627,495117 |
| 0000747_01 | 800 | UK | G51D carrier | #4 | DSG | 0.25 | 1 | 60 | 12304,66833 |
| 0000747_01 | 800 | UK | G51D carrier | #4 | DSG | 0.25 | 1 | 14 | 4302,099121 |
| 0000747_01 | 800 | UK | G51D carrier | #4 | DSG | 0.25 | 2 | 60 | 12043,71948 |
| 0000747_01 | 800 | UK | G51D carrier | #4 | DSG | 0.25 | 2 | 14 | 3824,730957 |
| 0000747_01 | 800 | UK | G51D carrier | #4 | DSG | 1.43 | 1 | 60 | 14512,12988 |
| 0000747_01 | 800 | UK | G51D carrier | #4 | DSG | 1.43 | 1 | 14 | 1364,953613 |
| 0000747_01 | 800 | UK | G51D carrier | #4 | DSG | 1.43 | 2 | 60 | 13290,75537 |
| 0000747_01 | 800 | UK | G51D carrier | #4 | DSG | 1.43 | 2 | 14 | 928,4360352 |
| 0000752_01 | 800 | UK | G51D carrier | #4 | GA | 0.0067 | 1 | 60 | 6272,182129 |
| 0000752_01 | 800 | UK | G51D carrier | #4 | GA | 0.0067 | 1 | 14 | 1263,869141 |
| 0000752_01 | 800 | UK | G51D carrier | #4 | GA | 0.0067 | 2 | 60 | 6070,058594 |
| 0000752_01 | 800 | UK | G51D carrier | #4 | GA | 0.0067 | 2 | 14 | 1557,327637 |
| 0000752_01 | 800 | UK | G51D carrier | #4 |  | 0 |  | 60 | 1024,32373 |
| 0000752_01 | 800 | UK | G51D carrier | #4 |  | 0 |  | 14 | 6666,66748 |
| 0000747_01 | 800 | UK | G51D carrier | #4 |  | 0 |  | 60 | 2038,857178 |
| 0000747_01 | 800 | UK | G51D carrier | #4 |  | 0 |  | 14 | 3835,453125 |
| 0000752_01 | 800 | UK | G51D carrier | #5 | DSG | 0.25 | 1 | 60 | 5429,755371 |
| 0000752_01 | 800 | UK | G51D carrier | #5 | DSG | 0.25 | 1 | 14 | 4503,956543 |
| 0000752_01 | 800 | UK | G51D carrier | #5 | DSG | 0.25 | 2 | 60 | 5301,407227 |
| 0000752_01 | 800 | UK | G51D carrier | #5 | DSG | 0.25 | 2 | 14 | 6905,626953 |
| 0001134_01 | 800 | UK | G51D carrier | #5 | DSG | 1.43 | 1 | 60 | 387,7231445 |
| 0001134_01 | 800 | UK | G51D carrier | #5 | DSG | 1.43 | 1 | 14 | 256,293457 |
| 0001134_01 | 800 | UK | G51D carrier | #5 | DSG | 1.43 | 2 | 60 | 697,1103516 |
| 0001134_01 | 800 | UK | G51D carrier | #5 | DSG | 1.43 | 2 | 14 | 552,034668 |
| 0000752_01 | 800 | UK | G51D carrier | #5 | GA | 0.0067 | 1 | 60 | 6611,186035 |
| 0000752_01 | 800 | UK | G51D carrier | #5 | GA | 0.0067 | 1 | 14 | 2705,494141 |
| 0000752_01 | 800 | UK | G51D carrier | #5 | GA | 0.0067 | 2 | 60 | 6490,452148 |
| 0000752_01 | 800 | UK | G51D carrier | #5 | GA | 0.0067 | 2 | 14 | 2527,335938 |
| 0000752_01 | 800 | UK | G51D carrier | #5 |  | 0 |  | 60 | 1392,041016 |
| 0000752_01 | 800 | UK | G51D carrier | #5 |  | 0 |  | 14 | 5086,996582 |
| 0000747_01 | 800 | UK | PD G51D | #6 | DSG | 0.25 | 1 | 60 | 6725,331055 |
| 0000747_01 | 800 | UK | PD G51D | #6 | DSG | 0.25 | 1 | 14 | 6413,87207 |
| 0000747_01 | 800 | UK | PD G51D | #6 | DSG | 0.25 | 2 | 60 | 6401,568604 |
| 0000747_01 | 800 | UK | PD G51D | #6 | DSG | 0.25 | 2 | 14 | 9069,680664 |
| 0000747_01 | 800 | UK | PD G51D | #6 | DSG | 1.43 | 1 | 60 | 8391,895752 |
| 0000747_01 | 800 | UK | PD G51D | #6 | DSG | 1.43 | 1 | 14 | 1996,37207 |
| 0000747_01 | 800 | UK | PD G51D | #6 | DSG | 1.43 | 2 | 60 | 7593,403809 |
| 0000747_01 | 800 | UK | PD G51D | #6 | DSG | 1.43 | 2 | 14 | 2310,820313 |
| 0000752_01 | 800 | UK | PD G51D | #6 | GA | 0.0067 | 1 | 60 | 5053,295898 |
| 0000752_01 | 800 | UK | PD G51D | #6 | GA | 0.0067 | 1 | 14 | 2872,213379 |
| 0000752_01 | 800 | UK | PD G51D | #6 | GA | 0.0067 | 2 | 60 | 4556,818359 |
| 0000752_01 | 800 | UK | PD G51D | #6 | GA | 0.0067 | 2 | 14 | 3436,65625 |
| 0000752_01 | 800 | UK | PD G51D | #6 |  | 0 |  | 60 | 518,0415039 |
| 0000752_01 | 800 | UK | PD G51D | #6 |  | 0 |  | 14 | 10243,80225 |
| 0000747_01 | 800 | UK | PD G51D | #6 |  | 0 |  | 60 | 977,5371094 |
| 0000747_01 | 800 | UK | PD G51D | #6 |  | 0 |  | 14 | 5792,041504 |

DSG=Disuccinimidyl glutarate, GA=glutaraldehyde; f.c.=final concentration (DSG in mM, GA in %), kDA=kilo Dalton, PD=Parkinson’s disease
