# Supplementary material for: Aggregation-resistant alpha-synuclein tetramers are reduced in the blood of Parkinson’s patients
Source: EMBO Mol Med. 2024 Jun 5;16(7):10. doi: 10.1038/s44321-024-00083-5 (PMC11250827; doi:10.1038/s44321-024-00083-5)
Supplement: Supplementary file 4 — Source data Fig. 3 [file 44321_2024_83_MOESM4_ESM.zip › Source Data Fig. 3/Fig. 3 Source Data.docx]

Summary of raw signal intensities from the LI-COR Image Studio Software for Fig. 3

| Image Name | Channel | Cohort | Classification | Subject | Crosslinker | Crosslinker f.c. | Technical replicate | kDa band | Signal intensity |
| --- | --- | --- | --- | --- | --- | --- | --- | --- | --- |
| 0001092_01 | 800 | UK | sPD | 1 | DSG | 1.43 | 1 | 60 | 814,45703 |
| 0001092_01 | 800 | UK | sPD | 1 | DSG | 1.43 | 1 | 14 | 2837,78418 |
| 0001092_01 | 800 | UK | sPD | 1 | DSG | 1.43 | 2 | 60 | 758,23584 |
| 0001092_01 | 800 | UK | sPD | 1 | DSG | 1.43 | 2 | 14 | 3809,79150 |
| 0001092_01 | 800 | UK | sPD | 2 | DSG | 1.43 | 1 | 60 | 913,84668 |
| 0001092_01 | 800 | UK | sPD | 2 | DSG | 1.43 | 1 | 14 | 3973,99707 |
| 0001092_01 | 800 | UK | sPD | 2 | DSG | 1.43 | 2 | 60 | 586,37451 |
| 0001092_01 | 800 | UK | sPD | 2 | DSG | 1.43 | 2 | 14 | 5093,40820 |
| 0001092_01 | 800 | UK | sPD | 3 | DSG | 1.43 | 1 | 60 | 533,53564 |
| 0001092_01 | 800 | UK | sPD | 3 | DSG | 1.43 | 1 | 14 | 2297,00781 |
| 0001092_01 | 800 | UK | sPD | 3 | DSG | 1.43 | 2 | 60 | 856,80273 |
| 0001092_01 | 800 | UK | sPD | 3 | DSG | 1.43 | 2 | 14 | 2899,39355 |
| 0001092_01 | 800 | UK | sPD | 4 | DSG | 1.43 | 1 | 60 | 957,47754 |
| 0001092_01 | 800 | UK | sPD | 4 | DSG | 1.43 | 1 | 14 | 1740,12305 |
| 0001092_01 | 800 | UK | sPD | 4 | DSG | 1.43 | 2 | 60 | 1054,52246 |
| 0001092_01 | 800 | UK | sPD | 4 | DSG | 1.43 | 2 | 14 | 1889,71143 |
| 0001092_01 | 800 | UK | sPD | 5 | DSG | 1.43 | 1 | 60 | 1301,24414 |
| 0001092_01 | 800 | UK | sPD | 5 | DSG | 1.43 | 1 | 14 | 10816,65527 |
| 0001092_01 | 800 | UK | sPD | 5 | DSG | 1.43 | 2 | 60 | 1111,13330 |
| 0001092_01 | 800 | UK | sPD | 5 | DSG | 1.43 | 2 | 14 | 5313,10645 |
| 0001092_01 | 800 | UK | sPD | 6 | DSG | 1.43 | 1 | 60 | 1500,64600 |
| 0001092_01 | 800 | UK | sPD | 6 | DSG | 1.43 | 1 | 14 | 3663,72900 |
| 0001092_01 | 800 | UK | sPD | 6 | DSG | 1.43 | 2 | 60 | 1250,31934 |
| 0001092_01 | 800 | UK | sPD | 6 | DSG | 1.43 | 2 | 14 | 5533,57031 |
| 0001091_01 | 800 | UK | sPD | 7 | DSG | 1.43 | 1 | 60 | 1126,16992 |
| 0001091_01 | 800 | UK | sPD | 7 | DSG | 1.43 | 1 | 14 | 3349,35156 |
| 0001091_01 | 800 | UK | sPD | 7 | DSG | 1.43 | 2 | 60 | 111,79297 |
| 0001091_01 | 800 | UK | sPD | 7 | DSG | 1.43 | 2 | 14 | 1966,18225 |
| 0001131_01 | 800 | UK | sPD | 8 | DSG | 1.43 | 1 | 60 | 633,0898438 |
| 0001131_01 | 800 | UK | sPD | 8 | DSG | 1.43 | 1 | 14 | 266,151123 |
| 0001131_01 | 800 | UK | sPD | 8 | DSG | 1.43 | 2 | 60 | 1148,213379 |
| 0001131_01 | 800 | UK | sPD | 8 | DSG | 1.43 | 2 | 14 | 189,3491211 |
| 0001134_01 | 800 | UK | sPD | 9 | DSG | 1.43 | 1 | 60 | 823,6992188 |
| 0001134_01 | 800 | UK | sPD | 9 | DSG | 1.43 | 1 | 14 | 1377,770996 |
| 0001134_01 | 800 | UK | sPD | 9 | DSG | 1.43 | 2 | 60 | 752,380127 |
| 0001134_01 | 800 | UK | sPD | 9 | DSG | 1.43 | 2 | 14 | 1555,164063 |
| 0001134_01 | 800 | UK | sPD | 9 | DSG | 1.44 | 3 | 60 | 311,7478027 |
| 0001134_01 | 800 | UK | sPD | 9 | DSG | 1.45 | 3 | 14 | 587,3994141 |
| 0001130_01 | 800 | UK | sPD | 10 | DSG | 1.43 | 1 | 60 | 530,2060547 |
| 0001130_01 | 800 | UK | sPD | 10 | DSG | 1.43 | 1 | 14 | 650,934082 |
| 0001130_01 | 800 | UK | sPD | 10 | DSG | 1.43 | 2 | 60 | 871,3686523 |
| 0001130_01 | 800 | UK | sPD | 10 | DSG | 1.43 | 2 | 14 | 1763,880371 |
| 0000461_01 | 800 | UK | sPD | 11 | DSG | 1.43 | 1 | 60 | 3578,345703 |
| 0000461_01 | 800 | UK | sPD | 11 | DSG | 1.43 | 1 | 14 | 884,4736328 |
| 0000461_01 | 800 | UK | sPD | 11 | DSG | 1.43 | 2 | 60 | 4597,543945 |
| 0000461_01 | 800 | UK | sPD | 11 | DSG | 1.43 | 2 | 14 | 832,2207031 |
| 0000461_01 | 800 | UK | sPD | 11 | DSG | 1.43 | 3 | 60 | 4038,613281 |
| 0000461_01 | 800 | UK | sPD | 11 | DSG | 1.43 | 3 | 14 | 967,09375 |
| 0000461_01 | 800 | UK | sPD | 12 | DSG | 1.43 | 1 | 60 | 4006,744141 |
| 0000461_01 | 800 | UK | sPD | 12 | DSG | 1.43 | 1 | 14 | 1130,163086 |
| 0000461_01 | 800 | UK | sPD | 12 | DSG | 1.43 | 2 | 60 | 4904,774414 |
| 0000461_01 | 800 | UK | sPD | 12 | DSG | 1.43 | 2 | 14 | 1417,227539 |
| 0000461_01 | 800 | UK | sPD | 12 | DSG | 1.43 | 3 | 60 | 4887,195313 |
| 0000461_01 | 800 | UK | sPD | 12 | DSG | 1.43 | 3 | 14 | 862,4570313 |
| 0000461_01 | 800 | UK | sPD | 13 | DSG | 1.43 | 1 | 60 | 4075,460938 |
| 0000461_01 | 800 | UK | sPD | 13 | DSG | 1.43 | 1 | 14 | 1435,032227 |
| 0000461_01 | 800 | UK | sPD | 13 | DSG | 1.43 | 2 | 60 | 3522,692383 |
| 0000461_01 | 800 | UK | sPD | 13 | DSG | 1.43 | 2 | 14 | 1763,075195 |
| 0000461_01 | 800 | UK | sPD | 13 | DSG | 1.43 | 3 | 60 | 4786,603516 |
| 0000461_01 | 800 | UK | sPD | 13 | DSG | 1.43 | 3 | 14 | 2845,144531 |
| 0000461_01 | 800 | UK | sPD | 14 | DSG | 1.43 | 1 | 60 | 2906,926758 |
| 0000461_01 | 800 | UK | sPD | 14 | DSG | 1.43 | 1 | 14 | 2793,488281 |
| 0000461_01 | 800 | UK | sPD | 14 | DSG | 1.43 | 2 | 60 | 3225,412598 |
| 0000461_01 | 800 | UK | sPD | 14 | DSG | 1.43 | 2 | 14 | 2762,301758 |
| 0000461_01 | 800 | UK | sPD | 14 | DSG | 1.43 | 3 | 60 | 2763,291992 |
| 0000461_01 | 800 | UK | sPD | 14 | DSG | 1.43 | 3 | 14 | 2410,224609 |
| 0000461_01 | 800 | UK | sPD | 15 | DSG | 1.43 | 1 | 60 | 3184,643066 |
| 0000461_01 | 800 | UK | sPD | 15 | DSG | 1.43 | 1 | 14 | 1954,231445 |
| 0000461_01 | 800 | UK | sPD | 15 | DSG | 1.43 | 2 | 60 | 3261,642578 |
| 0000461_01 | 800 | UK | sPD | 15 | DSG | 1.43 | 2 | 14 | 1907,007813 |
| 0000461_01 | 800 | UK | sPD | 15 | DSG | 1.43 | 3 | 60 | 3291,717285 |
| 0000461_01 | 800 | UK | sPD | 15 | DSG | 1.43 | 3 | 14 | 2207,643555 |
| 0000519_01 | 800 | UK | sPD | 16 | DSG | 1.43 | 1 | 60 | 5843,946289 |
| 0000519_01 | 800 | UK | sPD | 16 | DSG | 1.43 | 1 | 14 | 9202,941895 |
| 0000519_01 | 800 | UK | sPD | 16 | DSG | 1.43 | 2 | 60 | 6792,286377 |
| 0000519_01 | 800 | UK | sPD | 16 | DSG | 1.43 | 2 | 14 | 9686,868164 |
| 0000519_01 | 800 | UK | sPD | 16 | DSG | 1.43 | 3 | 60 | 7760,973938 |
| 0000519_01 | 800 | UK | sPD | 16 | DSG | 1.43 | 3 | 14 | 8565,984375 |
| 0000400_01 | 800 | UK | sPD | 17 | DSG | 1.43 | 1 | 60 | 562,166748 |
| 0000400_01 | 800 | UK | sPD | 17 | DSG | 1.43 | 1 | 14 | 184,4526367 |
| 0000400_01 | 800 | UK | sPD | 17 | DSG | 1.43 | 2 | 60 | 571,2988281 |
| 0000400_01 | 800 | UK | sPD | 17 | DSG | 1.43 | 2 | 14 | 339,5749512 |
| 0000400_01 | 800 | UK | sPD | 17 | DSG | 1.43 | 3 | 60 | 589,0776367 |
| 0000400_01 | 800 | UK | sPD | 17 | DSG | 1.43 | 3 | 14 | 257,0559082 |
| 0000400_01 | 800 | UK | sPD | 18 | DSG | 1.43 | 1 | 60 | 181,7980957 |
| 0000400_01 | 800 | UK | sPD | 18 | DSG | 1.43 | 1 | 14 | 210,0029297 |
| 0000400_01 | 800 | UK | sPD | 18 | DSG | 1.43 | 2 | 60 | 169,6223145 |
| 0000400_01 | 800 | UK | sPD | 18 | DSG | 1.43 | 2 | 14 | 148,3154297 |
| 0000400_01 | 800 | UK | sPD | 18 | DSG | 1.43 | 3 | 60 | 297,8056641 |
| 0000400_01 | 800 | UK | sPD | 18 | DSG | 1.43 | 3 | 14 | 245,467041 |
| 0000400_01 | 800 | UK | sPD | 19 | DSG | 1.43 | 1 | 60 | 334,6130371 |
| 0000400_01 | 800 | UK | sPD | 19 | DSG | 1.43 | 1 | 14 | 126,6394043 |
| 0000400_01 | 800 | UK | sPD | 19 | DSG | 1.43 | 2 | 60 | 318,3952637 |
| 0000400_01 | 800 | UK | sPD | 19 | DSG | 1.43 | 2 | 14 | 85,11767578 |
| 0000400_01 | 800 | UK | sPD | 19 | DSG | 1.43 | 3 | 60 | 370,020752 |
| 0000400_01 | 800 | UK | sPD | 19 | DSG | 1.43 | 3 | 14 | 122,2709961 |
| 0000400_01 | 800 | UK | sPD | 20 | DSG | 1.43 | 1 | 60 | 934,2607422 |
| 0000400_01 | 800 | UK | sPD | 20 | DSG | 1.43 | 1 | 14 | 567,6855469 |
| 0000400_01 | 800 | UK | sPD | 20 | DSG | 1.43 | 2 | 60 | 1124,071289 |
| 0000400_01 | 800 | UK | sPD | 20 | DSG | 1.43 | 2 | 14 | 471,4404297 |
| 0000400_01 | 800 | UK | sPD | 20 | DSG | 1.43 | 3 | 60 | 478,1281738 |
| 0000400_01 | 800 | UK | sPD | 20 | DSG | 1.43 | 3 | 14 | 210,5170898 |
| 0000400_01 | 800 | UK | sPD | 21 | DSG | 1.43 | 1 | 60 | 691,6313477 |
| 0000400_01 | 800 | UK | sPD | 21 | DSG | 1.43 | 1 | 14 | 593,7119141 |
| 0000400_01 | 800 | UK | sPD | 21 | DSG | 1.43 | 2 | 60 | 724,0134277 |
| 0000400_01 | 800 | UK | sPD | 21 | DSG | 1.43 | 2 | 14 | 297,1813965 |
| 0000400_01 | 800 | UK | sPD | 21 | DSG | 1.43 | 3 | 60 | 372,0224609 |
| 0000400_01 | 800 | UK | sPD | 21 | DSG | 1.43 | 3 | 14 | 253,1630859 |
| 0000400_01 | 800 | UK | sPD | 22 | DSG | 1.43 | 1 | 60 | 651,8671875 |
| 0000400_01 | 800 | UK | sPD | 22 | DSG | 1.43 | 1 | 14 | 672,8989258 |
| 0000400_01 | 800 | UK | sPD | 22 | DSG | 1.43 | 2 | 60 | 636,6506348 |
| 0000400_01 | 800 | UK | sPD | 22 | DSG | 1.43 | 2 | 14 | 630,2214355 |
| 0000400_01 | 800 | UK | sPD | 22 | DSG | 1.43 | 3 | 60 | 358,3132324 |
| 0000400_01 | 800 | UK | sPD | 22 | DSG | 1.43 | 3 | 14 | 266,1833496 |
| 0000400_01 | 800 | UK | sPD | 23 | DSG | 1.43 | 1 | 60 | 443,9052734 |
| 0000400_01 | 800 | UK | sPD | 23 | DSG | 1.43 | 1 | 14 | 165,8571777 |
| 0000400_01 | 800 | UK | sPD | 23 | DSG | 1.43 | 2 | 60 | 370,7614746 |
| 0000400_01 | 800 | UK | sPD | 23 | DSG | 1.43 | 2 | 14 | 193,7553711 |
| 0000400_01 | 800 | UK | sPD | 23 | DSG | 1.43 | 3 | 60 | 397,4641113 |
| 0000400_01 | 800 | UK | sPD | 23 | DSG | 1.43 | 3 | 14 | 232,7243652 |
| 0000400_01 | 800 | UK | sPD | 24 | DSG | 1.43 | 1 | 60 | 915,5498047 |
| 0000400_01 | 800 | UK | sPD | 24 | DSG | 1.43 | 1 | 14 | 209,8166504 |
| 0000400_01 | 800 | UK | sPD | 24 | DSG | 1.43 | 2 | 60 | 836,8959961 |
| 0000400_01 | 800 | UK | sPD | 24 | DSG | 1.43 | 2 | 14 | 196,8771973 |
| 0000400_01 | 800 | UK | sPD | 24 | DSG | 1.43 | 3 | 60 | 382,260498 |
| 0000400_01 | 800 | UK | sPD | 24 | DSG | 1.43 | 3 | 14 | 212,2861328 |
| 0000400_01 | 800 | UK | sPD | 25 | DSG | 1.43 | 1 | 60 | 1193,447754 |
| 0000400_01 | 800 | UK | sPD | 25 | DSG | 1.43 | 1 | 14 | 482,6416016 |
| 0000400_01 | 800 | UK | sPD | 25 | DSG | 1.43 | 2 | 60 | 1276,635254 |
| 0000400_01 | 800 | UK | sPD | 25 | DSG | 1.43 | 2 | 14 | 403,4494629 |
| 0000400_01 | 800 | UK | sPD | 25 | DSG | 1.43 | 3 | 60 | 362,2902832 |
| 0000400_01 | 800 | UK | sPD | 25 | DSG | 1.43 | 3 | 14 | 113,9611816 |
| 0000400_01 | 800 | UK | sPD | 26 | DSG | 1.43 | 1 | 60 | 1310,965576 |
| 0000400_01 | 800 | UK | sPD | 26 | DSG | 1.43 | 1 | 14 | 938,5175781 |
| 0000400_01 | 800 | UK | sPD | 26 | DSG | 1.43 | 2 | 60 | 1284,641846 |
| 0000400_01 | 800 | UK | sPD | 26 | DSG | 1.43 | 2 | 14 | 1086,309814 |
| 0000400_01 | 800 | UK | sPD | 26 | DSG | 1.43 | 3 | 60 | 483,4436035 |
| 0000400_01 | 800 | UK | sPD | 26 | DSG | 1.43 | 3 | 14 | 411,9641113 |
| 0000400_01 | 800 | UK | sPD | 27 | DSG | 1.43 | 1 | 60 | 1070,314453 |
| 0000400_01 | 800 | UK | sPD | 27 | DSG | 1.43 | 1 | 14 | 533,7507324 |
| 0000400_01 | 800 | UK | sPD | 27 | DSG | 1.43 | 2 | 60 | 1056,733887 |
| 0000400_01 | 800 | UK | sPD | 27 | DSG | 1.43 | 2 | 14 | 843,4111328 |
| 0000400_01 | 800 | UK | sPD | 27 | DSG | 1.43 | 3 | 60 | 359,8376465 |
| 0000400_01 | 800 | UK | sPD | 27 | DSG | 1.43 | 3 | 14 | 146,1330566 |
| 0000400_01 | 800 | UK | sPD | 28 | DSG | 1.43 | 1 | 60 | 1037,415771 |
| 0000400_01 | 800 | UK | sPD | 28 | DSG | 1.43 | 1 | 14 | 599,8701172 |
| 0000400_01 | 800 | UK | sPD | 28 | DSG | 1.43 | 2 | 60 | 1098,286865 |
| 0000400_01 | 800 | UK | sPD | 28 | DSG | 1.43 | 2 | 14 | 1176,450684 |
| 0000400_01 | 800 | UK | sPD | 28 | DSG | 1.43 | 3 | 60 | 343,1315918 |
| 0000400_01 | 800 | UK | sPD | 28 | DSG | 1.43 | 3 | 14 | 199,3227539 |
| 0000400_01 | 800 | UK | sPD | 29 | DSG | 1.43 | 1 | 60 | 778,7451172 |
| 0000400_01 | 800 | UK | sPD | 29 | DSG | 1.43 | 1 | 14 | 489,1669922 |
| 0000400_01 | 800 | UK | sPD | 29 | DSG | 1.43 | 2 | 60 | 932,7060547 |
| 0000400_01 | 800 | UK | sPD | 29 | DSG | 1.43 | 2 | 14 | 413,7177734 |
| 0000400_01 | 800 | UK | sPD | 29 | DSG | 1.43 | 3 | 60 | 371,6394043 |
| 0000400_01 | 800 | UK | sPD | 29 | DSG | 1.43 | 3 | 14 | 195,0419922 |
| 0000400_01 | 800 | UK | sPD | 30 | DSG | 1.43 | 1 | 60 | 1031,438965 |
| 0000400_01 | 800 | UK | sPD | 30 | DSG | 1.43 | 1 | 14 | 431,2043457 |
| 0000400_01 | 800 | UK | sPD | 30 | DSG | 1.43 | 2 | 60 | 1035,201172 |
| 0000400_01 | 800 | UK | sPD | 30 | DSG | 1.43 | 2 | 14 | 326,6501465 |
| 0000401_01 | 800 | UK | sPD | 30 | DSG | 1.43 | 3 | 60 | 409,5131836 |
| 0000401_01 | 800 | UK | sPD | 30 | DSG | 1.43 | 3 | 14 | 114,9313965 |
| 0000401_01 | 800 | UK | sPD | 31 | DSG | 1.43 | 1 | 60 | 821,9384766 |
| 0000401_01 | 800 | UK | sPD | 31 | DSG | 1.43 | 1 | 14 | 514,3283691 |
| 0000401_01 | 800 | UK | sPD | 31 | DSG | 1.43 | 2 | 60 | 772,2055664 |
| 0000401_01 | 800 | UK | sPD | 31 | DSG | 1.43 | 2 | 14 | 207,3032227 |
| 0000401_01 | 800 | UK | sPD | 31 | DSG | 1.43 | 3 | 60 | 350,7939453 |
| 0000401_01 | 800 | UK | sPD | 31 | DSG | 1.43 | 3 | 14 | 124,3903809 |
| 0000401_01 | 800 | UK | sPD | 32 | DSG | 1.43 | 1 | 60 | 960,7253418 |
| 0000401_01 | 800 | UK | sPD | 32 | DSG | 1.43 | 1 | 14 | 372,644043 |
| 0000401_01 | 800 | UK | sPD | 32 | DSG | 1.43 | 2 | 60 | 880,4628906 |
| 0000401_01 | 800 | UK | sPD | 32 | DSG | 1.43 | 2 | 14 | 288,3930664 |
| 0000401_01 | 800 | UK | sPD | 32 | DSG | 1.43 | 3 | 60 | 346,4238281 |
| 0000401_01 | 800 | UK | sPD | 32 | DSG | 1.43 | 3 | 14 | 189,7211914 |
| 0000401_01 | 800 | UK | sPD | 33 | DSG | 1.43 | 1 | 60 | 380,9567871 |
| 0000401_01 | 800 | UK | sPD | 33 | DSG | 1.43 | 1 | 14 | 110,2602539 |
| 0000401_01 | 800 | UK | sPD | 33 | DSG | 1.43 | 2 | 60 | 399,0981445 |
| 0000401_01 | 800 | UK | sPD | 33 | DSG | 1.43 | 2 | 14 | 159,3059082 |
| 0000401_01 | 800 | UK | sPD | 33 | DSG | 1.43 | 3 | 60 | 400,0429688 |
| 0000401_01 | 800 | UK | sPD | 33 | DSG | 1.43 | 3 | 14 | 118,0310059 |
| 0000401_01 | 800 | UK | sPD | 34 | DSG | 1.43 | 1 | 60 | 556,4365234 |
| 0000401_01 | 800 | UK | sPD | 34 | DSG | 1.43 | 1 | 14 | 201,184082 |
| 0000401_01 | 800 | UK | sPD | 34 | DSG | 1.43 | 2 | 60 | 582,420166 |
| 0000401_01 | 800 | UK | sPD | 34 | DSG | 1.43 | 2 | 14 | 330,4013672 |
| 0000401_01 | 800 | UK | sPD | 34 | DSG | 1.43 | 3 | 60 | 208,8671875 |
| 0000401_01 | 800 | UK | sPD | 34 | DSG | 1.43 | 3 | 14 | 218,6535645 |
| 0000401_01 | 800 | UK | sPD | 35 | DSG | 1.43 | 1 | 60 | 301,8161621 |
| 0000401_01 | 800 | UK | sPD | 35 | DSG | 1.43 | 1 | 14 | 313,5935059 |
| 0000401_01 | 800 | UK | sPD | 35 | DSG | 1.43 | 2 | 60 | 282,1079102 |
| 0000401_01 | 800 | UK | sPD | 35 | DSG | 1.43 | 2 | 14 | 363,0195313 |
| 0000401_01 | 800 | UK | sPD | 35 | DSG | 1.43 | 3 | 60 | 313,0822754 |
| 0000401_01 | 800 | UK | sPD | 35 | DSG | 1.43 | 3 | 14 | 362,9645996 |
| 0000401_01 | 800 | UK | sPD | 36 | DSG | 1.43 | 1 | 60 | 524,0209961 |
| 0000401_01 | 800 | UK | sPD | 36 | DSG | 1.43 | 1 | 14 | 214,8432617 |
| 0000401_01 | 800 | UK | sPD | 36 | DSG | 1.43 | 2 | 60 | 590,3051758 |
| 0000401_01 | 800 | UK | sPD | 36 | DSG | 1.43 | 2 | 14 | 221,0146484 |
| 0000401_01 | 800 | UK | sPD | 36 | DSG | 1.43 | 3 | 60 | 231,4033203 |
| 0000401_01 | 800 | UK | sPD | 36 | DSG | 1.43 | 3 | 14 | 165,9372559 |
| 0000401_01 | 800 | UK | sPD | 37 | DSG | 1.43 | 1 | 60 | 640,392334 |
| 0000401_01 | 800 | UK | sPD | 37 | DSG | 1.43 | 1 | 14 | 228,8557129 |
| 0000401_01 | 800 | UK | sPD | 37 | DSG | 1.43 | 2 | 60 | 509,7145996 |
| 0000401_01 | 800 | UK | sPD | 37 | DSG | 1.43 | 2 | 14 | 197,4399414 |
| 0000401_01 | 800 | UK | sPD | 37 | DSG | 1.43 | 3 | 60 | 249,8952637 |
| 0000401_01 | 800 | UK | sPD | 37 | DSG | 1.43 | 3 | 14 | 123,7075195 |
| 0000401_01 | 800 | UK | sPD | 38 | DSG | 1.43 | 1 | 60 | 396,4414063 |
| 0000401_01 | 800 | UK | sPD | 38 | DSG | 1.43 | 1 | 14 | 434,2612305 |
| 0000401_01 | 800 | UK | sPD | 38 | DSG | 1.43 | 2 | 60 | 518,9365234 |
| 0000401_01 | 800 | UK | sPD | 38 | DSG | 1.43 | 2 | 14 | 498,3789063 |
| 0000401_01 | 800 | UK | sPD | 38 | DSG | 1.43 | 3 | 60 | 225,0395508 |
| 0000401_01 | 800 | UK | sPD | 38 | DSG | 1.43 | 3 | 14 | 251,6149902 |
| 0000401_01 | 800 | UK | sPD | 39 | DSG | 1.43 | 1 | 60 | 490,1601563 |
| 0000401_01 | 800 | UK | sPD | 39 | DSG | 1.43 | 1 | 14 | 266,8662109 |
| 0000401_01 | 800 | UK | sPD | 39 | DSG | 1.43 | 2 | 60 | 580,25 |
| 0000401_01 | 800 | UK | sPD | 39 | DSG | 1.43 | 2 | 14 | 358,777832 |
| 0000401_01 | 800 | UK | sPD | 39 | DSG | 1.43 | 3 | 60 | 249,09375 |
| 0000401_01 | 800 | UK | sPD | 39 | DSG | 1.43 | 3 | 14 | 214,6003418 |
| 0000401_01 | 800 | UK | sPD | 40 | DSG | 1.43 | 1 | 60 | 359,0168457 |
| 0000401_01 | 800 | UK | sPD | 40 | DSG | 1.43 | 1 | 14 | 127,6452637 |
| 0000401_01 | 800 | UK | sPD | 40 | DSG | 1.43 | 2 | 60 | 357,5004883 |
| 0000401_01 | 800 | UK | sPD | 40 | DSG | 1.43 | 2 | 14 | 145,5461426 |
| 0000401_01 | 800 | UK | sPD | 40 | DSG | 1.43 | 3 | 60 | 358,5539551 |
| 0000401_01 | 800 | UK | sPD | 40 | DSG | 1.43 | 3 | 14 | 129,0141602 |
| 0000401_01 | 800 | UK | sPD | 41 | DSG | 1.43 | 1 | 60 | 494,4331055 |
| 0000401_01 | 800 | UK | sPD | 41 | DSG | 1.43 | 1 | 14 | 597,3984375 |
| 0000401_01 | 800 | UK | sPD | 41 | DSG | 1.43 | 2 | 60 | 542,8776855 |
| 0000401_01 | 800 | UK | sPD | 41 | DSG | 1.43 | 2 | 14 | 551,5366211 |
| 0000401_01 | 800 | UK | sPD | 41 | DSG | 1.43 | 3 | 60 | 248,208252 |
| 0000401_01 | 800 | UK | sPD | 41 | DSG | 1.43 | 3 | 14 | 325,878418 |
| 0000401_01 | 800 | UK | sPD | 42 | DSG | 1.43 | 1 | 60 | 511,4230957 |
| 0000401_01 | 800 | UK | sPD | 42 | DSG | 1.43 | 1 | 14 | 452,9648438 |
| 0000401_01 | 800 | UK | sPD | 42 | DSG | 1.43 | 2 | 60 | 576,2480469 |
| 0000401_01 | 800 | UK | sPD | 42 | DSG | 1.43 | 2 | 14 | 585,692627 |
| 0000401_01 | 800 | UK | sPD | 42 | DSG | 1.43 | 3 | 60 | 235,9960938 |
| 0000401_01 | 800 | UK | sPD | 42 | DSG | 1.43 | 3 | 14 | 192,7678223 |
| 0000401_01 | 800 | UK | sPD | 43 | DSG | 1.43 | 1 | 60 | 449,3476563 |
| 0000401_01 | 800 | UK | sPD | 43 | DSG | 1.43 | 1 | 14 | 646,1638184 |
| 0000401_01 | 800 | UK | sPD | 43 | DSG | 1.43 | 2 | 60 | 304,4067383 |
| 0000401_01 | 800 | UK | sPD | 43 | DSG | 1.43 | 2 | 14 | 568,4929199 |
| 0000401_01 | 800 | UK | sPD | 43 | DSG | 1.43 | 3 | 60 | 206,958252 |
| 0000401_01 | 800 | UK | sPD | 43 | DSG | 1.43 | 3 | 14 | 222,8935547 |
| 0000401_01 | 800 | UK | sPD | 44 | DSG | 1.43 | 1 | 60 | 435,0961914 |
| 0000401_01 | 800 | UK | sPD | 44 | DSG | 1.43 | 1 | 14 | 181,0458984 |
| 0000401_01 | 800 | UK | sPD | 44 | DSG | 1.43 | 2 | 60 | 533,222168 |
| 0000401_01 | 800 | UK | sPD | 44 | DSG | 1.43 | 2 | 14 | 299,5041504 |
| 0000401_01 | 800 | UK | sPD | 44 | DSG | 1.43 | 3 | 60 | 186,9187012 |
| 0000401_01 | 800 | UK | sPD | 44 | DSG | 1.43 | 3 | 14 | 102,1101074 |
| 0000401_01 | 800 | UK | sPD | 45 | DSG | 1.43 | 1 | 60 | 500,638916 |
| 0000401_01 | 800 | UK | sPD | 45 | DSG | 1.43 | 1 | 14 | 393,4206543 |
| 0000401_01 | 800 | UK | sPD | 45 | DSG | 1.43 | 2 | 60 | 608,6259766 |
| 0000401_01 | 800 | UK | sPD | 45 | DSG | 1.43 | 2 | 14 | 550,7167969 |
| 0000401_01 | 800 | UK | sPD | 45 | DSG | 1.43 | 3 | 60 | 142,5058594 |
| 0000401_01 | 800 | UK | sPD | 45 | DSG | 1.43 | 3 | 14 | 547,3664551 |
| 0000401_01 | 800 | UK | sPD | 46 | DSG | 1.43 | 1 | 60 | 531,5397949 |
| 0000401_01 | 800 | UK | sPD | 46 | DSG | 1.43 | 1 | 14 | 473,5317383 |
| 0000401_01 | 800 | UK | sPD | 46 | DSG | 1.43 | 2 | 60 | 466,4672852 |
| 0000401_01 | 800 | UK | sPD | 46 | DSG | 1.43 | 2 | 14 | 438,3071289 |
| 0000401_01 | 800 | UK | sPD | 46 | DSG | 1.43 | 3 | 60 | 200,0666504 |
| 0000401_01 | 800 | UK | sPD | 46 | DSG | 1.43 | 3 | 14 | 178,432373 |
| 0000401_01 | 800 | UK | sPD | 47 | DSG | 1.43 | 1 | 60 | 421,3391113 |
| 0000401_01 | 800 | UK | sPD | 47 | DSG | 1.43 | 1 | 14 | 228,5744629 |
| 0000401_01 | 800 | UK | sPD | 47 | DSG | 1.43 | 2 | 60 | 649,8017578 |
| 0000401_01 | 800 | UK | sPD | 47 | DSG | 1.43 | 2 | 14 | 365,7392578 |
| 0000401_01 | 800 | UK | sPD | 47 | DSG | 1.43 | 3 | 60 | 182,2609863 |
| 0000401_01 | 800 | UK | sPD | 47 | DSG | 1.43 | 3 | 14 | 115,8828125 |
| 0000401_01 | 800 | UK | sPD | 48 | DSG | 1.43 | 1 | 60 | 450,8039551 |
| 0000401_01 | 800 | UK | sPD | 48 | DSG | 1.43 | 1 | 14 | 277,807373 |
| 0000401_01 | 800 | UK | sPD | 48 | DSG | 1.43 | 2 | 60 | 485,7485352 |
| 0000401_01 | 800 | UK | sPD | 48 | DSG | 1.43 | 2 | 14 | 343,7392578 |
| 0000401_01 | 800 | UK | sPD | 48 | DSG | 1.43 | 3 | 60 | 204,9318848 |
| 0000401_01 | 800 | UK | sPD | 48 | DSG | 1.43 | 3 | 14 | 159,2792969 |
| 0000401_01 | 800 | UK | sPD | 49 | DSG | 1.43 | 1 | 60 | 632,0961914 |
| 0000401_01 | 800 | UK | sPD | 49 | DSG | 1.43 | 1 | 14 | 746,9189453 |
| 0000401_01 | 800 | UK | sPD | 49 | DSG | 1.43 | 2 | 60 | 606,409668 |
| 0000401_01 | 800 | UK | sPD | 49 | DSG | 1.43 | 2 | 14 | 719,0222168 |
| 0000401_01 | 800 | UK | sPD | 49 | DSG | 1.43 | 3 | 60 | 241,3520508 |
| 0000401_01 | 800 | UK | sPD | 49 | DSG | 1.43 | 3 | 14 | 428,479248 |
| 0000401_01 | 800 | UK | sPD | 50 | DSG | 1.43 | 1 | 60 | 318,8884277 |
| 0000401_01 | 800 | UK | sPD | 50 | DSG | 1.43 | 1 | 14 | 229,7189941 |
| 0000401_01 | 800 | UK | sPD | 50 | DSG | 1.43 | 2 | 60 | 279,4858398 |
| 0000401_01 | 800 | UK | sPD | 50 | DSG | 1.43 | 2 | 14 | 242,0725098 |
| 0000401_01 | 800 | UK | sPD | 50 | DSG | 1.43 | 3 | 60 | 206,4233398 |
| 0000401_01 | 800 | UK | sPD | 50 | DSG | 1.43 | 3 | 14 | 129,3786621 |
| 0000401_01 | 800 | UK | sPD | 51 | DSG | 1.43 | 1 | 60 | 341,0725098 |
| 0000401_01 | 800 | UK | sPD | 51 | DSG | 1.43 | 1 | 14 | 213,9265137 |
| 0000401_01 | 800 | UK | sPD | 51 | DSG | 1.43 | 2 | 60 | 364,2548828 |
| 0000401_01 | 800 | UK | sPD | 51 | DSG | 1.43 | 2 | 14 | 136,4187012 |
| 0000401_01 | 800 | UK | sPD | 51 | DSG | 1.43 | 3 | 60 | 198,7043457 |
| 0000401_01 | 800 | UK | sPD | 51 | DSG | 1.43 | 3 | 14 | 110,4816895 |
| 0000401_01 | 800 | UK | sPD | 52 | DSG | 1.43 | 1 | 60 | 304,9291992 |
| 0000401_01 | 800 | UK | sPD | 52 | DSG | 1.43 | 1 | 14 | 219,9248047 |
| 0000401_01 | 800 | UK | sPD | 52 | DSG | 1.43 | 2 | 60 | 313,9321289 |
| 0000401_01 | 800 | UK | sPD | 52 | DSG | 1.43 | 2 | 14 | 226,6755371 |
| 0000401_01 | 800 | UK | sPD | 52 | DSG | 1.43 | 3 | 60 | 80,47973633 |
| 0000401_01 | 800 | UK | sPD | 52 | DSG | 1.43 | 3 | 14 | 145,6420898 |
| 0000401_01 | 800 | UK | sPD | 53 | DSG | 1.43 | 1 | 60 | 547,8696289 |
| 0000401_01 | 800 | UK | sPD | 53 | DSG | 1.43 | 1 | 14 | 174,4543457 |
| 0000401_01 | 800 | UK | sPD | 53 | DSG | 1.43 | 2 | 60 | 580,2397461 |
| 0000401_01 | 800 | UK | sPD | 53 | DSG | 1.43 | 2 | 14 | 143,7302246 |
| 0000401_01 | 800 | UK | sPD | 53 | DSG | 1.43 | 3 | 60 | 641,7441406 |
| 0000401_01 | 800 | UK | sPD | 53 | DSG | 1.43 | 3 | 14 | 201,5192871 |
| 0000401_01 | 800 | UK | sPD | 54 | DSG | 1.43 | 1 | 60 | 425,1967773 |
| 0000401_01 | 800 | UK | sPD | 54 | DSG | 1.43 | 1 | 14 | 232,8669434 |
| 0000401_01 | 800 | UK | sPD | 54 | DSG | 1.43 | 2 | 60 | 416,6682129 |
| 0000401_01 | 800 | UK | sPD | 54 | DSG | 1.43 | 2 | 14 | 182,9562988 |
| 0000401_01 | 800 | UK | sPD | 54 | DSG | 1.43 | 3 | 60 | 727,4780273 |
| 0000401_01 | 800 | UK | sPD | 54 | DSG | 1.43 | 3 | 14 | 236,2631836 |
| 0000401_01 | 800 | UK | sPD | 55 | DSG | 1.43 | 1 | 60 | 706,7912598 |
| 0000401_01 | 800 | UK | sPD | 55 | DSG | 1.43 | 1 | 14 | 457,3686523 |
| 0000401_01 | 800 | UK | sPD | 55 | DSG | 1.43 | 2 | 60 | 781,9394531 |
| 0000401_01 | 800 | UK | sPD | 55 | DSG | 1.43 | 2 | 14 | 428,0109863 |
| 0001160_01 | 800 | UK | sPD | 55 | DSG | 1.43 | 3 | 60 | 799,9931641 |
| 0001160_01 | 800 | UK | sPD | 55 | DSG | 1.43 | 3 | 14 | 265,2109375 |
| 0001160_01 | 800 | UK | sPD | 56 | DSG | 1.43 | 1 | 60 | 1025,911133 |
| 0001160_01 | 800 | UK | sPD | 56 | DSG | 1.43 | 1 | 14 | 5846,119141 |
| 0001160_01 | 800 | UK | sPD | 56 | DSG | 1.43 | 2 | 60 | 519,0361328 |
| 0001160_01 | 800 | UK | sPD | 56 | DSG | 1.43 | 2 | 14 | 6481,413086 |
| 0001160_01 | 800 | UK | sPD | 57 | DSG | 1.43 | 1 | 60 | 1167,083984 |
| 0001160_01 | 800 | UK | sPD | 57 | DSG | 1.43 | 1 | 14 | 5095,537109 |
| 0001159_01 | 800 | UK | sPD | 57 | DSG | 1.43 | 2 | 60 | 1230,78125 |
| 0001159_01 | 800 | UK | sPD | 57 | DSG | 1.43 | 2 | 14 | 5152,493164 |
| 0001159_01 | 800 | UK | sPD | 58 | DSG | 1.43 | 1 | 60 | 732,4560547 |
| 0001159_01 | 800 | UK | sPD | 58 | DSG | 1.43 | 1 | 14 | 10749,59229 |
| 0001162_01 | 800 | UK | sPD | 58 | DSG | 1.43 | 2 | 60 | 1233,72168 |
| 0001162_01 | 800 | UK | sPD | 58 | DSG | 1.43 | 2 | 14 | 10315,94775 |
| 0001162_01 | 800 | UK | sPD | 59 | DSG | 1.43 | 1 | 60 | 431,3774414 |
| 0001162_01 | 800 | UK | sPD | 59 | DSG | 1.43 | 1 | 14 | 1831,356445 |
| 0001159_01 | 800 | UK | sPD | 59 | DSG | 1.43 | 2 | 60 | 222,5126648 |
| 0001159_01 | 800 | UK | sPD | 59 | DSG | 1.43 | 2 | 14 | 1281,873352 |
| 0001159_01 | 800 | UK | sPD | 60 | DSG | 1.43 | 1 | 60 | 947,5219727 |
| 0001159_01 | 800 | UK | sPD | 60 | DSG | 1.43 | 1 | 14 | 17178,05566 |
| 0000774_01 | 800 | UK | sPD | 60 | DSG | 1.43 | 2 | 60 | 1226,483398 |
| 0000774_01 | 800 | UK | sPD | 60 | DSG | 1.43 | 2 | 14 | 15386,68799 |
| 0000774_01 | 800 | UK | sPD | 61 | DSG | 1.43 | 1 | 60 | 2444,53125 |
| 0000774_01 | 800 | UK | sPD | 61 | DSG | 1.43 | 1 | 14 | 1123,599609 |
| 0000774_01 | 800 | UK | sPD | 61 | DSG | 1.43 | 2 | 60 | 2746,634277 |
| 0000774_01 | 800 | UK | sPD | 61 | DSG | 1.43 | 2 | 14 | 1352,273438 |
| 0000774_01 | 800 | UK | sPD | 62 | DSG | 1.43 | 1 | 60 | 3562,535156 |
| 0000774_01 | 800 | UK | sPD | 62 | DSG | 1.43 | 1 | 14 | 920,65625 |
| 0000774_01 | 800 | UK | sPD | 62 | DSG | 1.43 | 2 | 60 | 3756,413086 |
| 0000774_01 | 800 | UK | sPD | 62 | DSG | 1.43 | 2 | 14 | 784,6318359 |
| 0000774_01 | 800 | UK | sPD | 63 | DSG | 1.43 | 1 | 60 | 4116,035156 |
| 0000774_01 | 800 | UK | sPD | 63 | DSG | 1.43 | 1 | 14 | 2659,792969 |
| 0000774_01 | 800 | UK | sPD | 63 | DSG | 1.43 | 2 | 60 | 3388,193848 |
| 0000774_01 | 800 | UK | sPD | 63 | DSG | 1.43 | 2 | 14 | 2549,579102 |
| 0000774_01 | 800 | UK | sPD | 64 | DSG | 1.43 | 1 | 60 | 3339,867188 |
| 0000774_01 | 800 | UK | sPD | 64 | DSG | 1.43 | 1 | 14 | 1526,078613 |
| 0000774_01 | 800 | UK | sPD | 64 | DSG | 1.43 | 2 | 60 | 2767,614258 |
| 0000774_01 | 800 | UK | sPD | 64 | DSG | 1.43 | 2 | 14 | 1116,956543 |
| 0000461_01 | 800 | UK | sPD | 65 | DSG | 1.43 | 1 | 60 | 4331,690918 |
| 0000461_01 | 800 | UK | sPD | 65 | DSG | 1.43 | 1 | 14 | 3435,579102 |
| 0000461_01 | 800 | UK | sPD | 65 | DSG | 1.43 | 2 | 60 | 4200,383301 |
| 0000461_01 | 800 | UK | sPD | 65 | DSG | 1.43 | 2 | 14 | 3380,301758 |
| 0000461_01 | 800 | UK | sPD | 65 | DSG | 1.43 | 3 | 60 | 4221,029785 |
| 0000461_01 | 800 | UK | sPD | 65 | DSG | 1.43 | 3 | 14 | 3854,859375 |
| 0001130_01 | 800 | UK | Control | 1 | DSG | 1.43 | 1 | 60 | 857,99072 |
| 0001130_01 | 800 | UK | Control | 1 | DSG | 1.43 | 1 | 14 | 549,80811 |
| 0001130_01 | 800 | UK | Control | 1 | DSG | 1.43 | 2 | 60 | 743,47852 |
| 0001130_01 | 800 | UK | Control | 1 | DSG | 1.43 | 2 | 14 | 399,98145 |
| 0001130_01 | 800 | UK | Control | 2 | DSG | 1.43 | 1 | 60 | 504,07959 |
| 0001130_01 | 800 | UK | Control | 2 | DSG | 1.43 | 1 | 14 | 361,11328 |
| 0000461_01 | 800 | UK | Control | 2 | DSG | 1.43 | 2 | 60 | 448,25732 |
| 0000461_01 | 800 | UK | Control | 2 | DSG | 1.43 | 2 | 14 | 297,01904 |
| 0000461_01 | 800 | UK | Control | 3 | DSG | 1.43 | 1 | 60 | 4782,984375 |
| 0000461_01 | 800 | UK | Control | 3 | DSG | 1.43 | 1 | 14 | 2968,532227 |
| 0000461_01 | 800 | UK | Control | 3 | DSG | 1.43 | 2 | 60 | 3767,112305 |
| 0000461_01 | 800 | UK | Control | 3 | DSG | 1.43 | 2 | 14 | 3458,466797 |
| 0000461_01 | 800 | UK | Control | 3 | DSG | 1.43 | 3 | 60 | 4030,742188 |
| 0000461_01 | 800 | UK | Control | 3 | DSG | 1.43 | 3 | 14 | 2578,585938 |
| 0000461_01 | 800 | UK | Control | 4 | DSG | 1.43 | 1 | 60 | 3078,293457 |
| 0000461_01 | 800 | UK | Control | 4 | DSG | 1.43 | 1 | 14 | 1657,984375 |
| 0000461_01 | 800 | UK | Control | 4 | DSG | 1.43 | 2 | 60 | 3439,424805 |
| 0000461_01 | 800 | UK | Control | 4 | DSG | 1.43 | 2 | 14 | 2335,086914 |
| 0000400_01 | 800 | UK | Control | 4 | DSG | 1.43 | 3 | 60 | 2997,089844 |
| 0000400_01 | 800 | UK | Control | 4 | DSG | 1.43 | 3 | 14 | 2154,547852 |
| 0000400_01 | 800 | UK | Control | 5 | DSG | 1.43 | 1 | 60 | 848,5732422 |
| 0000400_01 | 800 | UK | Control | 5 | DSG | 1.43 | 1 | 14 | 340,8503418 |
| 0000400_01 | 800 | UK | Control | 5 | DSG | 1.43 | 2 | 60 | 859,6801758 |
| 0000400_01 | 800 | UK | Control | 5 | DSG | 1.43 | 2 | 14 | 508,1083984 |
| 0000400_01 | 800 | UK | Control | 5 | DSG | 1.43 | 3 | 60 | 392,9897461 |
| 0000400_01 | 800 | UK | Control | 5 | DSG | 1.43 | 3 | 14 | 171,6179199 |
| 0000400_01 | 800 | UK | Control | 6 | DSG | 1.43 | 1 | 60 | 897,559082 |
| 0000400_01 | 800 | UK | Control | 6 | DSG | 1.43 | 1 | 14 | 293,1491699 |
| 0000400_01 | 800 | UK | Control | 6 | DSG | 1.43 | 2 | 60 | 833,215332 |
| 0000400_01 | 800 | UK | Control | 6 | DSG | 1.43 | 2 | 14 | 292,1884766 |
| 0000401_01 | 800 | UK | Control | 6 | DSG | 1.43 | 3 | 60 | 360,8186035 |
| 0000401_01 | 800 | UK | Control | 6 | DSG | 1.43 | 3 | 14 | 175,541748 |
| 0000401_01 | 800 | UK | Control | 7 | DSG | 1.43 | 1 | 60 | 701,53125 |
| 0000401_01 | 800 | UK | Control | 7 | DSG | 1.43 | 1 | 14 | 152,0327148 |
| 0000401_01 | 800 | UK | Control | 7 | DSG | 1.43 | 2 | 60 | 660,3481445 |
| 0000401_01 | 800 | UK | Control | 7 | DSG | 1.43 | 2 | 14 | 318,4804688 |
| 0000401_01 | 800 | UK | Control | 7 | DSG | 1.43 | 3 | 60 | 215,220459 |
| 0000401_01 | 800 | UK | Control | 7 | DSG | 1.43 | 3 | 14 | 164,2438965 |
| 0000401_01 | 800 | UK | Control | 8 | DSG | 1.43 | 1 | 60 | 229,5041504 |
| 0000401_01 | 800 | UK | Control | 8 | DSG | 1.43 | 1 | 14 | 147,7370605 |
| 0000401_01 | 800 | UK | Control | 8 | DSG | 1.43 | 2 | 60 | 280,3212891 |
| 0000401_01 | 800 | UK | Control | 8 | DSG | 1.43 | 2 | 14 | 123,298584 |
| 0000401_01 | 800 | UK | Control | 8 | DSG | 1.43 | 3 | 60 | 256,7998047 |
| 0000401_01 | 800 | UK | Control | 8 | DSG | 1.43 | 3 | 14 | 126,239502 |
| 0000401_01 | 800 | UK | Control | 9 | DSG | 1.43 | 1 | 60 | 609,6044922 |
| 0000401_01 | 800 | UK | Control | 9 | DSG | 1.43 | 1 | 14 | 82,47387695 |
| 0000401_01 | 800 | UK | Control | 9 | DSG | 1.43 | 2 | 60 | 682,7741699 |
| 0000401_01 | 800 | UK | Control | 9 | DSG | 1.43 | 2 | 14 | 146,0407715 |
| 0000401_01 | 800 | UK | Control | 9 | DSG | 1.43 | 3 | 60 | 266,9892578 |
| 0000401_01 | 800 | UK | Control | 9 | DSG | 1.43 | 3 | 14 | 145,5361328 |
| 0000401_01 | 800 | UK | Control | 10 | DSG | 1.43 | 1 | 60 | 458,4165039 |
| 0000401_01 | 800 | UK | Control | 10 | DSG | 1.43 | 1 | 14 | 160,3359375 |
| 0000401_01 | 800 | UK | Control | 10 | DSG | 1.43 | 2 | 60 | 423,4143066 |
| 0000401_01 | 800 | UK | Control | 10 | DSG | 1.43 | 2 | 14 | 167,0412598 |
| 0000401_01 | 800 | UK | Control | 10 | DSG | 1.43 | 3 | 60 | 221,4467773 |
| 0000401_01 | 800 | UK | Control | 10 | DSG | 1.43 | 3 | 14 | 104,2314453 |
| 0000401_01 | 800 | UK | Control | 11 | DSG | 1.43 | 1 | 60 | 414,4926758 |
| 0000401_01 | 800 | UK | Control | 11 | DSG | 1.43 | 1 | 14 | 228,7900391 |
| 0000401_01 | 800 | UK | Control | 11 | DSG | 1.43 | 2 | 60 | 417,8039551 |
| 0000401_01 | 800 | UK | Control | 11 | DSG | 1.43 | 2 | 14 | 205,8068848 |
| 0000401_01 | 800 | UK | Control | 11 | DSG | 1.43 | 3 | 60 | 248,0891113 |
| 0000401_01 | 800 | UK | Control | 11 | DSG | 1.43 | 3 | 14 | 168,1945801 |
| 0000401_01 | 800 | UK | Control | 12 | DSG | 1.43 | 1 | 60 | 476,2983398 |
| 0000401_01 | 800 | UK | Control | 12 | DSG | 1.43 | 1 | 14 | 167,8850098 |
| 0000401_01 | 800 | UK | Control | 12 | DSG | 1.43 | 2 | 60 | 480,9194336 |
| 0000401_01 | 800 | UK | Control | 12 | DSG | 1.43 | 2 | 14 | 246,2026367 |
| 0000401_01 | 800 | UK | Control | 12 | DSG | 1.43 | 3 | 60 | 639,9841309 |
| 0000401_01 | 800 | UK | Control | 12 | DSG | 1.43 | 3 | 14 | 180,4643555 |
| 0000401_01 | 800 | UK | Control | 12 | DSG | 1.43 | 4 | 60 | 623,1010742 |
| 0000401_01 | 800 | UK | Control | 12 | DSG | 1.43 | 4 | 14 | 219,6513672 |
| 0000401_01 | 800 | UK | Control | 12 | DSG | 1.43 | 5 | 60 | 660,9233398 |
| 0000401_01 | 800 | UK | Control | 12 | DSG | 1.43 | 5 | 14 | 168,9511719 |
| 0000774_01 | 800 | UK | Control | 13 | DSG | 1.43 | 1 | 60 | 2870,579102 |
| 0000774_01 | 800 | UK | Control | 13 | DSG | 1.43 | 1 | 14 | 2148,625977 |
| 0000774_01 | 800 | UK | Control | 13 | DSG | 1.43 | 2 | 60 | 2931,79248 |
| 0000774_01 | 800 | UK | Control | 13 | DSG | 1.43 | 2 | 14 | 2376,416016 |
| 0000774_01 | 800 | UK | Control | 14 | DSG | 1.43 | 1 | 60 | 3822,963867 |
| 0000774_01 | 800 | UK | Control | 14 | DSG | 1.43 | 1 | 14 | 1030,478516 |
| 0000774_01 | 800 | UK | Control | 14 | DSG | 1.43 | 2 | 60 | 3476,838379 |
| 0000774_01 | 800 | UK | Control | 14 | DSG | 1.43 | 2 | 14 | 1042,406738 |
| 0000774_01 | 800 | UK | Control | 15 | DSG | 1.43 | 1 | 60 | 1910,080078 |
| 0000774_01 | 800 | UK | Control | 15 | DSG | 1.43 | 1 | 14 | 961,9521484 |
| 0000774_01 | 800 | UK | Control | 15 | DSG | 1.43 | 2 | 60 | 2059,488281 |
| 0000774_01 | 800 | UK | Control | 15 | DSG | 1.43 | 2 | 14 | 726,8388672 |
| 0000774_01 | 800 | UK | Control | 16 | DSG | 1.43 | 1 | 60 | 2937,914063 |
| 0000774_01 | 800 | UK | Control | 16 | DSG | 1.43 | 1 | 14 | 2510,001953 |
| 0000774_01 | 800 | UK | Control | 16 | DSG | 1.43 | 2 | 60 | 2413,024414 |
| 0000774_01 | 800 | UK | Control | 16 | DSG | 1.43 | 2 | 14 | 1557,776367 |
| 0000774_01 | 800 | UK | Control | 17 | DSG | 1.43 | 1 | 60 | 2084,943848 |
| 0000774_01 | 800 | UK | Control | 17 | DSG | 1.43 | 1 | 14 | 878,859375 |
| 0000774_01 | 800 | UK | Control | 17 | DSG | 1.43 | 2 | 60 | 2019,731445 |
| 0000774_01 | 800 | UK | Control | 17 | DSG | 1.43 | 2 | 14 | 998,2768555 |
| 0005859_02 | 800 | Germany | sPD | 1 | GA | 0.0067 | 1 | 60 | 943,7353516 |
| 0005859_02 | 800 | Germany | sPD | 1 | GA | 0.0067 | 1 | 14 | 1075,216797 |
| 0005859_02 | 800 | Germany | sPD | 1 | GA | 0.0067 | 2 | 60 | 645,567627 |
| 0005859_02 | 800 | Germany | sPD | 1 | GA | 0.0067 | 2 | 14 | 863,1088867 |
| 0005858_02 | 800 | Germany | sPD | 2 | GA | 0.0067 | 1 | 60 | 839,4951172 |
| 0005858_02 | 800 | Germany | sPD | 2 | GA | 0.0067 | 1 | 14 | 717,5595703 |
| 0005858_02 | 800 | Germany | sPD | 2 | GA | 0.0067 | 2 | 60 | 791,1416016 |
| 0005858_02 | 800 | Germany | sPD | 2 | GA | 0.0067 | 2 | 14 | 750,921875 |
| 0005858_02 | 800 | Germany | sPD | 3 | GA | 0.0067 | 1 | 60 | 1218,655762 |
| 0005858_02 | 800 | Germany | sPD | 3 | GA | 0.0067 | 1 | 14 | 1426,817871 |
| 0005858_02 | 800 | Germany | sPD | 3 | GA | 0.0067 | 2 | 60 | 1221,179199 |
| 0005858_02 | 800 | Germany | sPD | 3 | GA | 0.0067 | 2 | 14 | 1406,210449 |
| 0005858_02 | 800 | Germany | sPD | 4 | GA | 0.0067 | 1 | 60 | 985,0444336 |
| 0005858_02 | 800 | Germany | sPD | 4 | GA | 0.0067 | 1 | 14 | 351,7548828 |
| 0005858_02 | 800 | Germany | sPD | 4 | GA | 0.0067 | 2 | 60 | 994,8774414 |
| 0005858_02 | 800 | Germany | sPD | 4 | GA | 0.0067 | 2 | 14 | 406,796875 |
| 0005858_02 | 800 | Germany | sPD | 5 | GA | 0.0067 | 1 | 60 | 1202,156738 |
| 0005858_02 | 800 | Germany | sPD | 5 | GA | 0.0067 | 1 | 14 | 450,1230469 |
| 0005858_02 | 800 | Germany | sPD | 5 | GA | 0.0067 | 2 | 60 | 1112,430664 |
| 0005858_02 | 800 | Germany | sPD | 5 | GA | 0.0067 | 2 | 14 | 538,5605469 |
| 0006296_01 | 800 | Germany | sPD | 6 | GA | 0.0067 | 1 | 60 | 616,5654297 |
| 0006296_01 | 800 | Germany | sPD | 6 | GA | 0.0067 | 1 | 14 | 140,4296875 |
| 0006296_01 | 800 | Germany | sPD | 6 | GA | 0.0067 | 2 | 60 | 1466,795898 |
| 0006296_01 | 800 | Germany | sPD | 6 | GA | 0.0067 | 2 | 14 | 403,1757813 |
| 0006296_01 | 800 | Germany | sPD | 7 | GA | 0.0067 | 1 | 60 | 1160,952148 |
| 0006296_01 | 800 | Germany | sPD | 7 | GA | 0.0067 | 1 | 14 | 699,2822266 |
| 0006296_01 | 800 | Germany | sPD | 7 | GA | 0.0067 | 2 | 60 | 1503,104492 |
| 0006296_01 | 800 | Germany | sPD | 7 | GA | 0.0067 | 2 | 14 | 548,5058594 |
| 0006296_01 | 800 | Germany | sPD | 8 | GA | 0.0067 | 1 | 60 | 1376,344727 |
| 0006296_01 | 800 | Germany | sPD | 8 | GA | 0.0067 | 1 | 14 | 686,3554688 |
| 0006296_01 | 800 | Germany | sPD | 8 | GA | 0.0067 | 2 | 60 | 1563,354492 |
| 0006296_01 | 800 | Germany | sPD | 8 | GA | 0.0067 | 2 | 14 | 511,7724609 |
| 0006296_01 | 800 | Germany | sPD | 9 | GA | 0.0067 | 1 | 60 | 728,5605469 |
| 0006296_01 | 800 | Germany | sPD | 9 | GA | 0.0067 | 1 | 14 | 306,4482422 |
| 0006296_01 | 800 | Germany | sPD | 9 | GA | 0.0067 | 2 | 60 | 1713,291992 |
| 0006296_01 | 800 | Germany | sPD | 9 | GA | 0.0067 | 2 | 14 | 417,4755859 |
| 0006296_01 | 800 | Germany | sPD | 10 | GA | 0.0067 | 1 | 60 | 1763,032227 |
| 0006296_01 | 800 | Germany | sPD | 10 | GA | 0.0067 | 1 | 14 | 363,4091797 |
| 0006296_01 | 800 | Germany | sPD | 10 | GA | 0.0067 | 2 | 60 | 1785,985352 |
| 0006296_01 | 800 | Germany | sPD | 10 | GA | 0.0067 | 2 | 14 | 230,1191406 |
| 0006296_01 | 800 | Germany | sPD | 11 | GA | 0.0067 | 1 | 60 | 1081,952148 |
| 0006296_01 | 800 | Germany | sPD | 11 | GA | 0.0067 | 1 | 14 | 300,0732422 |
| 0006296_01 | 800 | Germany | sPD | 11 | GA | 0.0067 | 2 | 60 | 1223,098633 |
| 0006296_01 | 800 | Germany | sPD | 11 | GA | 0.0067 | 2 | 14 | 174,53125 |
| 0006296_01 | 800 | Germany | sPD | 12 | GA | 0.0067 | 1 | 60 | 1746,03125 |
| 0006296_01 | 800 | Germany | sPD | 12 | GA | 0.0067 | 1 | 14 | 388,9599609 |
| 0006296_01 | 800 | Germany | sPD | 12 | GA | 0.0067 | 2 | 60 | 1614,307617 |
| 0006296_01 | 800 | Germany | sPD | 12 | GA | 0.0067 | 2 | 14 | 490,9355469 |
| 0006296_01 | 800 | Germany | sPD | 13 | GA | 0.0067 | 1 | 60 | 1752,929688 |
| 0006296_01 | 800 | Germany | sPD | 13 | GA | 0.0067 | 1 | 14 | 479,8154297 |
| 0006296_01 | 800 | Germany | sPD | 13 | GA | 0.0067 | 2 | 60 | 1924,664063 |
| 0006296_01 | 800 | Germany | sPD | 13 | GA | 0.0067 | 2 | 14 | 543,0727539 |
| 0006296_01 | 800 | Germany | sPD | 14 | GA | 0.0067 | 1 | 60 | 1802,202148 |
| 0006296_01 | 800 | Germany | sPD | 14 | GA | 0.0067 | 1 | 14 | 304,2314453 |
| 0006296_01 | 800 | Germany | sPD | 14 | GA | 0.0067 | 2 | 60 | 1531,210938 |
| 0006296_01 | 800 | Germany | sPD | 14 | GA | 0.0067 | 2 | 14 | 479,5322266 |
| 0006296_01 | 800 | Germany | sPD | 15 | GA | 0.0067 | 1 | 60 | 1655,080078 |
| 0006296_01 | 800 | Germany | sPD | 15 | GA | 0.0067 | 1 | 14 | 731,3916016 |
| 0006296_01 | 800 | Germany | sPD | 15 | GA | 0.0067 | 2 | 60 | 1402,414063 |
| 0006296_01 | 800 | Germany | sPD | 15 | GA | 0.0067 | 2 | 14 | 681,1445313 |
| 0006296_01 | 800 | Germany | sPD | 16 | GA | 0.0067 | 1 | 60 | 757,1699219 |
| 0006296_01 | 800 | Germany | sPD | 16 | GA | 0.0067 | 1 | 14 | 344,7373047 |
| 0006296_01 | 800 | Germany | sPD | 16 | GA | 0.0067 | 2 | 60 | 823,4814453 |
| 0006296_01 | 800 | Germany | sPD | 16 | GA | 0.0067 | 2 | 14 | 351,4423828 |
| 0006296_01 | 800 | Germany | sPD | 17 | GA | 0.0067 | 1 | 60 | 1499,108398 |
| 0006296_01 | 800 | Germany | sPD | 17 | GA | 0.0067 | 1 | 14 | 417,9677734 |
| 0006296_01 | 800 | Germany | sPD | 17 | GA | 0.0067 | 2 | 60 | 1805,123047 |
| 0006296_01 | 800 | Germany | sPD | 17 | GA | 0.0067 | 2 | 14 | 504,90625 |
| 0006296_01 | 800 | Germany | sPD | 18 | GA | 0.0067 | 1 | 60 | 653,9814453 |
| 0006296_01 | 800 | Germany | sPD | 18 | GA | 0.0067 | 1 | 14 | 255,3828125 |
| 0006296_01 | 800 | Germany | sPD | 18 | GA | 0.0067 | 2 | 60 | 696,9448242 |
| 0006296_01 | 800 | Germany | sPD | 18 | GA | 0.0067 | 2 | 14 | 235,6488037 |
| 0006296_01 | 800 | Germany | sPD | 19 | GA | 0.0067 | 1 | 60 | 528,0634766 |
| 0006296_01 | 800 | Germany | sPD | 19 | GA | 0.0067 | 1 | 14 | 127,0224609 |
| 0006296_01 | 800 | Germany | sPD | 19 | GA | 0.0067 | 2 | 60 | 674,7939453 |
| 0006296_01 | 800 | Germany | sPD | 19 | GA | 0.0067 | 2 | 14 | 196,9453125 |
| 0006296_01 | 800 | Germany | sPD | 20 | GA | 0.0067 | 1 | 60 | 1026,352051 |
| 0006296_01 | 800 | Germany | sPD | 20 | GA | 0.0067 | 1 | 14 | 277,0878906 |
| 0006296_01 | 800 | Germany | sPD | 20 | GA | 0.0067 | 2 | 60 | 1082,181641 |
| 0006296_01 | 800 | Germany | sPD | 20 | GA | 0.0067 | 2 | 14 | 457,6933594 |
| 0006296_01 | 800 | Germany | sPD | 21 | GA | 0.0067 | 1 | 60 | 1370,017578 |
| 0006296_01 | 800 | Germany | sPD | 21 | GA | 0.0067 | 1 | 14 | 305,2363281 |
| 0006296_01 | 800 | Germany | sPD | 21 | GA | 0.0067 | 2 | 60 | 1303,611328 |
| 0006296_01 | 800 | Germany | sPD | 21 | GA | 0.0067 | 2 | 14 | 448,5761719 |
| 0006296_01 | 800 | Germany | sPD | 22 | GA | 0.0067 | 1 | 60 | 1111,644531 |
| 0006296_01 | 800 | Germany | sPD | 22 | GA | 0.0067 | 1 | 14 | 439,6748047 |
| 0006296_01 | 800 | Germany | sPD | 22 | GA | 0.0067 | 2 | 60 | 1049,986328 |
| 0006296_01 | 800 | Germany | sPD | 22 | GA | 0.0067 | 2 | 14 | 359,0566406 |
| 0006296_01 | 800 | Germany | sPD | 23 | GA | 0.0067 | 1 | 60 | 836,1953125 |
| 0006296_01 | 800 | Germany | sPD | 23 | GA | 0.0067 | 1 | 14 | 534,6796875 |
| 0006296_01 | 800 | Germany | sPD | 23 | GA | 0.0067 | 2 | 60 | 693,7304688 |
| 0006296_01 | 800 | Germany | sPD | 23 | GA | 0.0067 | 2 | 14 | 452,4619141 |
| 0006334_02 | 800 | Germany | sPD | 24 | GA | 0.0067 | 1 | 60 | 1117,586426 |
| 0006334_02 | 800 | Germany | sPD | 24 | GA | 0.0067 | 1 | 14 | 300,8955078 |
| 0006334_02 | 800 | Germany | sPD | 24 | GA | 0.0067 | 2 | 60 | 760,9990234 |
| 0006334_02 | 800 | Germany | sPD | 24 | GA | 0.0067 | 2 | 14 | 209,6518555 |
| 0006334_02 | 800 | Germany | sPD | 25 | GA | 0.0067 | 1 | 60 | 859,0581055 |
| 0006334_02 | 800 | Germany | sPD | 25 | GA | 0.0067 | 1 | 14 | 244,9750977 |
| 0006334_02 | 800 | Germany | sPD | 25 | GA | 0.0067 | 2 | 60 | 812,3535156 |
| 0006334_02 | 800 | Germany | sPD | 25 | GA | 0.0067 | 2 | 14 | 117,1904297 |
| 0006334_02 | 800 | Germany | sPD | 26 | GA | 0.0067 | 1 | 60 | 595,9609375 |
| 0006334_02 | 800 | Germany | sPD | 26 | GA | 0.0067 | 1 | 14 | 407,4404297 |
| 0006334_02 | 800 | Germany | sPD | 26 | GA | 0.0067 | 2 | 60 | 545,800293 |
| 0006334_02 | 800 | Germany | sPD | 26 | GA | 0.0067 | 2 | 14 | 129,9628906 |
| 0006334_02 | 800 | Germany | sPD | 27 | GA | 0.0067 | 1 | 60 | 719,9243164 |
| 0006334_02 | 800 | Germany | sPD | 27 | GA | 0.0067 | 1 | 14 | 166,6218262 |
| 0006334_02 | 800 | Germany | sPD | 27 | GA | 0.0067 | 2 | 60 | 627,7338867 |
| 0006334_02 | 800 | Germany | sPD | 27 | GA | 0.0067 | 2 | 14 | 137,4921875 |
| 0006334_02 | 800 | Germany | sPD | 28 | GA | 0.0067 | 1 | 60 | 427,4868164 |
| 0006334_02 | 800 | Germany | sPD | 28 | GA | 0.0067 | 1 | 14 | 73,90332031 |
| 0006334_02 | 800 | Germany | sPD | 28 | GA | 0.0067 | 2 | 60 | 524,3881836 |
| 0006334_02 | 800 | Germany | sPD | 28 | GA | 0.0067 | 2 | 14 | 132,3178711 |
| 0006334_02 | 800 | Germany | sPD | 29 | GA | 0.0067 | 1 | 60 | 599,3916016 |
| 0006334_02 | 800 | Germany | sPD | 29 | GA | 0.0067 | 1 | 14 | 155,7563477 |
| 0006334_02 | 800 | Germany | sPD | 29 | GA | 0.0067 | 2 | 60 | 578,2714844 |
| 0006334_02 | 800 | Germany | sPD | 29 | GA | 0.0067 | 2 | 14 | 169,7148438 |
| 0006334_02 | 800 | Germany | sPD | 30 | GA | 0.0067 | 1 | 60 | 576,7729492 |
| 0006334_02 | 800 | Germany | sPD | 30 | GA | 0.0067 | 1 | 14 | 290,0551758 |
| 0006334_02 | 800 | Germany | sPD | 30 | GA | 0.0067 | 2 | 60 | 496,4804688 |
| 0006334_02 | 800 | Germany | sPD | 30 | GA | 0.0067 | 2 | 14 | 278,5410156 |
| 0006334_02 | 800 | Germany | sPD | 31 | GA | 0.0067 | 1 | 60 | 496,0117188 |
| 0006334_02 | 800 | Germany | sPD | 31 | GA | 0.0067 | 1 | 14 | 145,5214844 |
| 0006334_02 | 800 | Germany | sPD | 31 | GA | 0.0067 | 2 | 60 | 527,3242188 |
| 0006334_02 | 800 | Germany | sPD | 31 | GA | 0.0067 | 2 | 14 | 154,5966797 |
| 0006334_02 | 800 | Germany | sPD | 32 | GA | 0.0067 | 1 | 60 | 586,65625 |
| 0006334_02 | 800 | Germany | sPD | 32 | GA | 0.0067 | 1 | 14 | 194,7514648 |
|  | 800 | Germany | sPD | 32 | GA | 0.0067 | 2 | 60 |  |
|  | 800 | Germany | sPD | 32 | GA | 0.0067 | 2 | 14 |  |
| 0006334_02 | 800 | Germany | sPD | 33 | GA | 0.0067 | 1 | 60 | 786,5639648 |
| 0006334_02 | 800 | Germany | sPD | 33 | GA | 0.0067 | 1 | 14 | 246,7636719 |
| 0006334_02 | 800 | Germany | sPD | 33 | GA | 0.0067 | 2 | 60 | 847,6948242 |
| 0006334_02 | 800 | Germany | sPD | 33 | GA | 0.0067 | 2 | 14 | 272,5454102 |
| 0006334_02 | 800 | Germany | sPD | 34 | GA | 0.0067 | 1 | 60 | 497,3237305 |
| 0006334_02 | 800 | Germany | sPD | 34 | GA | 0.0067 | 1 | 14 | 153,2946777 |
| 0006334_02 | 800 | Germany | sPD | 34 | GA | 0.0067 | 2 | 60 | 480,5756836 |
| 0006334_02 | 800 | Germany | sPD | 34 | GA | 0.0067 | 2 | 14 | 176,1962891 |
| 0006334_02 | 800 | Germany | sPD | 35 | GA | 0.0067 | 1 | 60 | 480,1884766 |
| 0006334_02 | 800 | Germany | sPD | 35 | GA | 0.0067 | 1 | 14 | 314,0712891 |
| 0006334_02 | 800 | Germany | sPD | 35 | GA | 0.0067 | 2 | 60 | 583,8530273 |
| 0006334_02 | 800 | Germany | sPD | 35 | GA | 0.0067 | 2 | 14 | 268,6499023 |
| 0006334_02 | 800 | Germany | sPD | 36 | GA | 0.0067 | 1 | 60 | 775,2763672 |
| 0006334_02 | 800 | Germany | sPD | 36 | GA | 0.0067 | 1 | 14 | 319,1147461 |
| 0006334_02 | 800 | Germany | sPD | 36 | GA | 0.0067 | 2 | 60 | 807,1967773 |
| 0006334_02 | 800 | Germany | sPD | 36 | GA | 0.0067 | 2 | 14 | 278,9482422 |
| 0006334_02 | 800 | Germany | sPD | 37 | GA | 0.0067 | 1 | 60 | 722,9204102 |
| 0006334_02 | 800 | Germany | sPD | 37 | GA | 0.0067 | 1 | 14 | 275,21875 |
| 0006334_02 | 800 | Germany | sPD | 37 | GA | 0.0067 | 2 | 60 | 684,7392578 |
| 0006334_02 | 800 | Germany | sPD | 37 | GA | 0.0067 | 2 | 14 | 182,6049805 |
| 0006371_02 | 800 | Germany | sPD | 38 | GA | 0.0067 | 1 | 60 | 1655,304199 |
| 0006371_02 | 800 | Germany | sPD | 38 | GA | 0.0067 | 1 | 14 | 325,2910156 |
| 0006371_02 | 800 | Germany | sPD | 38 | GA | 0.0067 | 2 | 60 | 1267,929688 |
| 0006371_02 | 800 | Germany | sPD | 38 | GA | 0.0067 | 2 | 14 | 362,9052734 |
| 0006371_02 | 800 | Germany | sPD | 39 | GA | 0.0067 | 1 | 60 | 922,4213867 |
| 0006371_02 | 800 | Germany | sPD | 39 | GA | 0.0067 | 1 | 14 | 295,0761719 |
| 0006371_02 | 800 | Germany | sPD | 39 | GA | 0.0067 | 2 | 60 | 971,0063477 |
| 0006371_02 | 800 | Germany | sPD | 39 | GA | 0.0067 | 2 | 14 | 520,4013672 |
| 0006371_02 | 800 | Germany | sPD | 40 | GA | 0.0067 | 1 | 60 | 582,6191406 |
| 0006371_02 | 800 | Germany | sPD | 40 | GA | 0.0067 | 1 | 14 | 100,1914063 |
| 0006371_02 | 800 | Germany | sPD | 40 | GA | 0.0067 | 2 | 60 | 618,5693359 |
| 0006371_02 | 800 | Germany | sPD | 40 | GA | 0.0067 | 2 | 14 | 108,6054688 |
| 0006371_02 | 800 | Germany | sPD | 41 | GA | 0.0067 | 1 | 60 | 968,1025391 |
| 0006371_02 | 800 | Germany | sPD | 41 | GA | 0.0067 | 1 | 14 | 177,9238281 |
| 0006371_02 | 800 | Germany | sPD | 41 | GA | 0.0067 | 2 | 60 | 742,9560547 |
| 0006371_02 | 800 | Germany | sPD | 41 | GA | 0.0067 | 2 | 14 | 306,7255859 |
| 0006371_02 | 800 | Germany | sPD | 42 | GA | 0.0067 | 1 | 60 | 927,7001953 |
| 0006371_02 | 800 | Germany | sPD | 42 | GA | 0.0067 | 1 | 14 | 394,5029297 |
| 0006371_02 | 800 | Germany | sPD | 42 | GA | 0.0067 | 2 | 60 | 1014,68457 |
| 0006371_02 | 800 | Germany | sPD | 42 | GA | 0.0067 | 2 | 14 | 513,9199219 |
| 0006371_02 | 800 | Germany | sPD | 43 | GA | 0.0067 | 1 | 60 | 1174,495605 |
| 0006371_02 | 800 | Germany | sPD | 43 | GA | 0.0067 | 1 | 14 | 133,1337891 |
| 0006371_02 | 800 | Germany | sPD | 43 | GA | 0.0067 | 2 | 60 | 1353,160645 |
| 0006371_02 | 800 | Germany | sPD | 43 | GA | 0.0067 | 2 | 14 | 157,3115234 |
| 0006371_02 | 800 | Germany | sPD | 44 | GA | 0.0067 | 1 | 60 | 1257,78418 |
| 0006371_02 | 800 | Germany | sPD | 44 | GA | 0.0067 | 1 | 14 | 171,6035156 |
| 0006371_02 | 800 | Germany | sPD | 44 | GA | 0.0067 | 2 | 60 | 1135,090332 |
| 0006371_02 | 800 | Germany | sPD | 44 | GA | 0.0067 | 2 | 14 | 321,3007813 |
| 0006371_02 | 800 | Germany | sPD | 45 | GA | 0.0067 | 1 | 60 | 808,1298828 |
| 0006371_02 | 800 | Germany | sPD | 45 | GA | 0.0067 | 1 | 14 | 153,3916016 |
| 0006371_02 | 800 | Germany | sPD | 45 | GA | 0.0067 | 2 | 60 | 768,3222656 |
| 0006371_02 | 800 | Germany | sPD | 45 | GA | 0.0067 | 2 | 14 | 108,1484375 |
| 0006371_02 | 800 | Germany | sPD | 46 | GA | 0.0067 | 1 | 60 | 1149,626953 |
| 0006371_02 | 800 | Germany | sPD | 46 | GA | 0.0067 | 1 | 14 | 384,6010742 |
| 0006371_02 | 800 | Germany | sPD | 46 | GA | 0.0067 | 2 | 60 | 554,8110352 |
| 0006371_02 | 800 | Germany | sPD | 46 | GA | 0.0067 | 2 | 14 | 468,2133789 |
| 0006371_02 | 800 | Germany | sPD | 47 | GA | 0.0067 | 1 | 60 | 1053,90625 |
| 0006371_02 | 800 | Germany | sPD | 47 | GA | 0.0067 | 1 | 14 | 248,2177734 |
| 0006371_02 | 800 | Germany | sPD | 47 | GA | 0.0067 | 2 | 60 | 960,2553711 |
| 0006371_02 | 800 | Germany | sPD | 47 | GA | 0.0067 | 2 | 14 | 198,6494141 |
| 0005882_02 | 800 | Germany | sPD | 48 | GA | 0.0067 | 1 | 60 | 312,9213867 |
| 0005882_02 | 800 | Germany | sPD | 48 | GA | 0.0067 | 1 | 14 | 86,72802734 |
| 0005882_02 | 800 | Germany | sPD | 48 | GA | 0.0067 | 2 | 60 | 281,5776367 |
| 0005882_02 | 800 | Germany | sPD | 48 | GA | 0.0067 | 2 | 14 | 74,93261719 |
| 0005882_02 | 800 | Germany | sPD | 49 | GA | 0.0067 | 1 | 60 | 356,6923828 |
| 0005882_02 | 800 | Germany | sPD | 49 | GA | 0.0067 | 1 | 14 | 273,9692383 |
| 0005882_02 | 800 | Germany | sPD | 49 | GA | 0.0067 | 2 | 60 | 414,8793945 |
| 0005882_02 | 800 | Germany | sPD | 49 | GA | 0.0067 | 2 | 14 | 217,9003906 |
| 0005882_02 | 800 | Germany | sPD | 50 | GA | 0.0067 | 1 | 60 | 433,3701172 |
| 0005882_02 | 800 | Germany | sPD | 50 | GA | 0.0067 | 1 | 14 | 118,7529297 |
| 0005882_02 | 800 | Germany | sPD | 50 | GA | 0.0067 | 2 | 60 | 372,5123291 |
| 0005882_02 | 800 | Germany | sPD | 50 | GA | 0.0067 | 2 | 14 | 114,267334 |
| 0005882_02 | 800 | Germany | sPD | 51 | GA | 0.0067 | 1 | 60 | 403,1660156 |
| 0005882_02 | 800 | Germany | sPD | 51 | GA | 0.0067 | 1 | 14 | 123,5385742 |
| 0005882_02 | 800 | Germany | sPD | 51 | GA | 0.0067 | 2 | 60 | 635,8867188 |
| 0005882_02 | 800 | Germany | sPD | 51 | GA | 0.0067 | 2 | 14 | 124,1821289 |
| 0005882_02 | 800 | Germany | sPD | 52 | GA | 0.0067 | 1 | 60 | 564,7910156 |
| 0005882_02 | 800 | Germany | sPD | 52 | GA | 0.0067 | 1 | 14 | 337,0668945 |
| 0005882_02 | 800 | Germany | sPD | 52 | GA | 0.0067 | 2 | 60 | 502,4282227 |
| 0005882_02 | 800 | Germany | sPD | 52 | GA | 0.0067 | 2 | 14 | 271,3442383 |
| 0005882_02 | 800 | Germany | sPD | 53 | GA | 0.0067 | 1 | 60 | 1223,11377 |
| 0005882_02 | 800 | Germany | sPD | 53 | GA | 0.0067 | 1 | 14 | 544,9282227 |
| 0005882_02 | 800 | Germany | sPD | 53 | GA | 0.0067 | 2 | 60 | 1736,939453 |
| 0005882_02 | 800 | Germany | sPD | 53 | GA | 0.0067 | 2 | 14 | 875,3833008 |
| 0005882_02 | 800 | Germany | sPD | 54 | GA | 0.0067 | 1 | 60 | 2357,234863 |
| 0005882_02 | 800 | Germany | sPD | 54 | GA | 0.0067 | 1 | 14 | 481,3369141 |
| 0005882_02 | 800 | Germany | sPD | 54 | GA | 0.0067 | 2 | 60 | 1704,178711 |
| 0005882_02 | 800 | Germany | sPD | 54 | GA | 0.0067 | 2 | 14 | 579,8901367 |
| 0005882_02 | 800 | Germany | sPD | 55 | GA | 0.0067 | 1 | 60 | 715,8837891 |
| 0005882_02 | 800 | Germany | sPD | 55 | GA | 0.0067 | 1 | 14 | 291,0429688 |
| 0005882_02 | 800 | Germany | sPD | 55 | GA | 0.0067 | 2 | 60 | 566,0004883 |
| 0005882_02 | 800 | Germany | sPD | 55 | GA | 0.0067 | 2 | 14 | 223,9365234 |
| 0005882_02 | 800 | Germany | sPD | 56 | GA | 0.0067 | 1 | 60 | 575,3325195 |
| 0005882_02 | 800 | Germany | sPD | 56 | GA | 0.0067 | 1 | 14 | 384,6279297 |
| 0005882_02 | 800 | Germany | sPD | 56 | GA | 0.0067 | 2 | 60 | 798,5512695 |
| 0005882_02 | 800 | Germany | sPD | 56 | GA | 0.0067 | 2 | 14 | 751,1674805 |
| 0005858_02 | 800 | Germany | sPD | 57 | GA | 0.0067 | 1 | 60 | 720,3525391 |
| 0005858_02 | 800 | Germany | sPD | 57 | GA | 0.0067 | 1 | 14 | 598,5039063 |
| 0005858_02 | 800 | Germany | sPD | 57 | GA | 0.0067 | 2 | 60 | 883,8334961 |
| 0005858_02 | 800 | Germany | sPD | 57 | GA | 0.0067 | 2 | 14 | 356,4106445 |
| 0005858_02 | 800 | Germany | sPD | 58 | GA | 0.0067 | 1 | 60 | 538,2861328 |
| 0005858_02 | 800 | Germany | sPD | 58 | GA | 0.0067 | 1 | 14 | 142,1313477 |
| 0005858_02 | 800 | Germany | sPD | 58 | GA | 0.0067 | 2 | 60 | 583,8955078 |
| 0005858_02 | 800 | Germany | sPD | 58 | GA | 0.0067 | 2 | 14 | 205,487793 |
| 0005858_02 | 800 | Germany | sPD | 59 | GA | 0.0067 | 1 | 60 | 1667,370117 |
| 0005858_02 | 800 | Germany | sPD | 59 | GA | 0.0067 | 1 | 14 | 456,2680664 |
| 0005858_02 | 800 | Germany | sPD | 59 | GA | 0.0067 | 2 | 60 | 1909,45459 |
| 0005858_02 | 800 | Germany | sPD | 59 | GA | 0.0067 | 2 | 14 | 489,8100586 |
| 0005858_02 | 800 | Germany | sPD | 60 | GA | 0.0067 | 1 | 60 | 1988,824219 |
| 0005858_02 | 800 | Germany | sPD | 60 | GA | 0.0067 | 1 | 14 | 1085,383789 |
| 0005858_02 | 800 | Germany | sPD | 60 | GA | 0.0067 | 2 | 60 | 1740,272461 |
| 0005858_02 | 800 | Germany | sPD | 60 | GA | 0.0067 | 2 | 14 | 785,0024414 |
| 0005858_02 | 800 | Germany | sPD | 61 | GA | 0.0067 | 1 | 60 | 1387,350098 |
| 0005858_02 | 800 | Germany | sPD | 61 | GA | 0.0067 | 1 | 14 | 547,3603516 |
| 0005858_02 | 800 | Germany | sPD | 61 | GA | 0.0067 | 2 | 60 | 1234,22998 |
| 0005858_02 | 800 | Germany | sPD | 61 | GA | 0.0067 | 2 | 14 | 654,4277344 |
| 0005858_02 | 800 | Germany | sPD | 62 | GA | 0.0067 | 1 | 60 | 1575,671387 |
| 0005858_02 | 800 | Germany | sPD | 62 | GA | 0.0067 | 1 | 14 | 780,6835938 |
| 0005858_02 | 800 | Germany | sPD | 62 | GA | 0.0067 | 2 | 60 | 1529,977783 |
| 0005858_02 | 800 | Germany | sPD | 62 | GA | 0.0067 | 2 | 14 | 957,0888672 |
| 0005882_02 | 800 | Germany | sPD | 63 | GA | 0.0067 | 1 | 60 | 545,3032227 |
| 0005882_02 | 800 | Germany | sPD | 63 | GA | 0.0067 | 1 | 14 | 152,6923828 |
| 0005882_02 | 800 | Germany | sPD | 63 | GA | 0.0067 | 2 | 60 | 795,8432617 |
| 0005882_02 | 800 | Germany | sPD | 63 | GA | 0.0067 | 2 | 14 | 238,3666992 |
| 0006334_02 | 800 | Germany | sPD | 64 | GA | 0.0067 | 1 | 60 | 784,4492188 |
| 0006334_02 | 800 | Germany | sPD | 64 | GA | 0.0067 | 1 | 14 | 67,07470703 |
| 0006334_02 | 800 | Germany | sPD | 64 | GA | 0.0067 | 2 | 60 | 738,5605469 |
| 0006334_02 | 800 | Germany | sPD | 64 | GA | 0.0067 | 2 | 14 | 150,1074219 |
| 0005857_02 | 800 | Germany | Control | 1 | GA | 0.0067 | 1 | 60 | 958,7709961 |
| 0005857_02 | 800 | Germany | Control | 1 | GA | 0.0067 | 1 | 14 | 165,5092773 |
| 0005857_02 | 800 | Germany | Control | 1 | GA | 0.0067 | 2 | 60 | 931,1450195 |
| 0005857_02 | 800 | Germany | Control | 1 | GA | 0.0067 | 2 | 14 | 182,1118164 |
| 0005857_02 | 800 | Germany | Control | 2 | GA | 0.0067 | 1 | 60 | 995,2749023 |
| 0005857_02 | 800 | Germany | Control | 2 | GA | 0.0067 | 1 | 14 | 228,5395508 |
| 0005857_02 | 800 | Germany | Control | 2 | GA | 0.0067 | 2 | 60 | 918,3466797 |
| 0005857_02 | 800 | Germany | Control | 2 | GA | 0.0067 | 2 | 14 | 294,1879883 |
| 0005857_02 | 800 | Germany | Control | 3 | GA | 0.0067 | 1 | 60 | 663,2988281 |
| 0005857_02 | 800 | Germany | Control | 3 | GA | 0.0067 | 1 | 14 | 446,9287109 |
| 0005857_02 | 800 | Germany | Control | 3 | GA | 0.0067 | 2 | 60 | 615,3203125 |
| 0005857_02 | 800 | Germany | Control | 3 | GA | 0.0067 | 2 | 14 | 360,2382813 |
| 0005857_02 | 800 | Germany | Control | 4 | GA | 0.0067 | 1 | 60 | 667,2602539 |
| 0005857_02 | 800 | Germany | Control | 4 | GA | 0.0067 | 1 | 14 | 327,0131836 |
| 0005857_02 | 800 | Germany | Control | 4 | GA | 0.0067 | 2 | 60 | 283,059082 |
| 0005857_02 | 800 | Germany | Control | 4 | GA | 0.0067 | 2 | 14 | 168,8164063 |
| 0005857_02 | 800 | Germany | Control | 5 | GA | 0.0067 | 1 | 60 | 800,3129883 |
| 0005857_02 | 800 | Germany | Control | 5 | GA | 0.0067 | 1 | 14 | 392,7866211 |
| 0005857_02 | 800 | Germany | Control | 5 | GA | 0.0067 | 2 | 60 | 704,9672852 |
| 0005857_02 | 800 | Germany | Control | 5 | GA | 0.0067 | 2 | 14 | 336,875 |
| 0005857_02 | 800 | Germany | Control | 6 | GA | 0.0067 | 1 | 60 | 768,1582031 |
| 0005857_02 | 800 | Germany | Control | 6 | GA | 0.0067 | 1 | 14 | 500,1640625 |
| 0005857_02 | 800 | Germany | Control | 6 | GA | 0.0067 | 2 | 60 | 738,3125 |
| 0005857_02 | 800 | Germany | Control | 6 | GA | 0.0067 | 2 | 14 | 462,7553711 |
| 0005857_02 | 800 | Germany | Control | 7 | GA | 0.0067 | 1 | 60 | 1283,015137 |
| 0005857_02 | 800 | Germany | Control | 7 | GA | 0.0067 | 1 | 14 | 272,8652344 |
| 0005857_02 | 800 | Germany | Control | 7 | GA | 0.0067 | 2 | 60 | 1308,806152 |
| 0005857_02 | 800 | Germany | Control | 7 | GA | 0.0067 | 2 | 14 | 288,5942383 |
| 0005857_02 | 800 | Germany | Control | 8 | GA | 0.0067 | 1 | 60 | 1414,138672 |
| 0005857_02 | 800 | Germany | Control | 8 | GA | 0.0067 | 1 | 14 | 162,0073242 |
| 0005857_02 | 800 | Germany | Control | 8 | GA | 0.0067 | 2 | 60 | 1300,029297 |
| 0005857_02 | 800 | Germany | Control | 8 | GA | 0.0067 | 2 | 14 | 126,2407227 |
| 0005857_02 | 800 | Germany | Control | 9 | GA | 0.0067 | 1 | 60 | 978,840332 |
| 0005857_02 | 800 | Germany | Control | 9 | GA | 0.0067 | 1 | 14 | 185,3291016 |
| 0005857_02 | 800 | Germany | Control | 9 | GA | 0.0067 | 2 | 60 | 401,9389648 |
| 0005857_02 | 800 | Germany | Control | 9 | GA | 0.0067 | 2 | 14 | 160,2675781 |
| 0005857_02 | 800 | Germany | Control | 10 | GA | 0.0067 | 1 | 60 | 103,4328613 |
| 0005857_02 | 800 | Germany | Control | 10 | GA | 0.0067 | 1 | 14 | 182,1025391 |
| 0005857_02 | 800 | Germany | Control | 10 | GA | 0.0067 | 2 | 60 | 937,8212891 |
| 0005857_02 | 800 | Germany | Control | 10 | GA | 0.0067 | 2 | 14 | 215,9833984 |
| 0005857_02 | 800 | Germany | Control | 11 | GA | 0.0067 | 1 | 60 | 1098,964355 |
| 0005857_02 | 800 | Germany | Control | 11 | GA | 0.0067 | 1 | 14 | 217,5415039 |
| 0005857_02 | 800 | Germany | Control | 11 | GA | 0.0067 | 2 | 60 | 1123,781738 |
| 0005857_02 | 800 | Germany | Control | 11 | GA | 0.0067 | 2 | 14 | 269,7216797 |
| 0005857_02 | 800 | Germany | Control | 12 | GA | 0.0067 | 1 | 60 | 996,0810547 |
| 0005857_02 | 800 | Germany | Control | 12 | GA | 0.0067 | 1 | 14 | 189,5722656 |
| 0005857_02 | 800 | Germany | Control | 12 | GA | 0.0067 | 2 | 60 | 819,6660156 |
| 0005857_02 | 800 | Germany | Control | 12 | GA | 0.0067 | 2 | 14 | 205,1552734 |
| 0005882_02 | 800 | Germany | Control | 13 | GA | 0.0067 | 1 | 60 | 1091,037109 |
| 0005882_02 | 800 | Germany | Control | 13 | GA | 0.0067 | 1 | 14 | 369,1040039 |
| 0005882_02 | 800 | Germany | Control | 13 | GA | 0.0067 | 2 | 60 | 793,7470703 |
| 0005882_02 | 800 | Germany | Control | 13 | GA | 0.0067 | 2 | 14 | 202,5019531 |
| 0005882_02 | 800 | Germany | Control | 14 | GA | 0.0067 | 1 | 60 | 750,1494141 |
| 0005882_02 | 800 | Germany | Control | 14 | GA | 0.0067 | 1 | 14 | 124,4238281 |
| 0005882_02 | 800 | Germany | Control | 14 | GA | 0.0067 | 2 | 60 | 395,5400391 |
| 0005882_02 | 800 | Germany | Control | 14 | GA | 0.0067 | 2 | 14 | 66,15722656 |
| 0005882_02 | 800 | Germany | Control | 15 | GA | 0.0067 | 1 | 60 | 2053,933594 |
| 0005882_02 | 800 | Germany | Control | 15 | GA | 0.0067 | 1 | 14 | 203,8510742 |
| 0005882_02 | 800 | Germany | Control | 15 | GA | 0.0067 | 2 | 60 | 991,15625 |
| 0005882_02 | 800 | Germany | Control | 15 | GA | 0.0067 | 2 | 14 | 70,55371094 |
| 0005882_02 | 800 | Germany | Control | 16 | GA | 0.0067 | 1 | 60 | 1164,773438 |
| 0005882_02 | 800 | Germany | Control | 16 | GA | 0.0067 | 1 | 14 | 80,95019531 |
| 0005882_02 | 800 | Germany | Control | 16 | GA | 0.0067 | 2 | 60 | 798,237793 |
| 0005882_02 | 800 | Germany | Control | 16 | GA | 0.0067 | 2 | 14 | 78,03759766 |
| 0005882_02 | 800 | Germany | Control | 17 | GA | 0.0067 | 1 | 60 | 810,9921875 |
| 0005882_02 | 800 | Germany | Control | 17 | GA | 0.0067 | 1 | 14 | 405,3100586 |
| 0005882_02 | 800 | Germany | Control | 17 | GA | 0.0067 | 2 | 60 | 605,9223633 |
| 0005882_02 | 800 | Germany | Control | 17 | GA | 0.0067 | 2 | 14 | 293,769043 |
| 0005882_02 | 800 | Germany | Control | 18 | GA | 0.0067 | 1 | 60 | 661,8789063 |
| 0005882_02 | 800 | Germany | Control | 18 | GA | 0.0067 | 1 | 14 | 254,6694336 |
| 0005882_02 | 800 | Germany | Control | 18 | GA | 0.0067 | 2 | 60 | 440,3564453 |
| 0005882_02 | 800 | Germany | Control | 18 | GA | 0.0067 | 2 | 14 | 186,065918 |
| 0005882_02 | 800 | Germany | Control | 19 | GA | 0.0067 | 1 | 60 | 251,478302 |
| 0005882_02 | 800 | Germany | Control | 19 | GA | 0.0067 | 1 | 14 | 37,95605469 |
| 0005882_02 | 800 | Germany | Control | 19 | GA | 0.0067 | 2 | 60 | 526,6884766 |
| 0005882_02 | 800 | Germany | Control | 19 | GA | 0.0067 | 2 | 14 | 62,32324219 |
| 0005882_02 | 800 | Germany | Control | 20 | GA | 0.0067 | 1 | 60 | 504,7519531 |
| 0005882_02 | 800 | Germany | Control | 20 | GA | 0.0067 | 1 | 14 | 121,3095703 |
| 0005882_02 | 800 | Germany | Control | 20 | GA | 0.0067 | 2 | 60 | 514,0800781 |
| 0005882_02 | 800 | Germany | Control | 20 | GA | 0.0067 | 2 | 14 | 93,96337891 |
| 0005882_02 | 800 | Germany | Control | 21 | GA | 0.0067 | 1 | 60 | 348,1865234 |
| 0005882_02 | 800 | Germany | Control | 21 | GA | 0.0067 | 1 | 14 | 111,8989258 |
| 0005882_02 | 800 | Germany | Control | 21 | GA | 0.0067 | 2 | 60 | 480,0844727 |
| 0005882_02 | 800 | Germany | Control | 21 | GA | 0.0067 | 2 | 14 | 133,5219727 |
| 0005882_02 | 800 | Germany | Control | 22 | GA | 0.0067 | 1 | 60 | 708,6845703 |
| 0005882_02 | 800 | Germany | Control | 22 | GA | 0.0067 | 1 | 14 | 246,1850586 |
| 0005882_02 | 800 | Germany | Control | 22 | GA | 0.0067 | 2 | 60 | 266,5625 |
| 0005882_02 | 800 | Germany | Control | 22 | GA | 0.0067 | 2 | 14 | 125,6464844 |
| 0005882_02 | 800 | Germany | Control | 23 | GA | 0.0067 | 1 | 60 | 330,8286133 |
| 0005882_02 | 800 | Germany | Control | 23 | GA | 0.0067 | 1 | 14 | 128,2983398 |
| 0005882_02 | 800 | Germany | Control | 23 | GA | 0.0067 | 2 | 60 | 296,90625 |
| 0005882_02 | 800 | Germany | Control | 23 | GA | 0.0067 | 2 | 14 | 100,7563477 |
| 0005882_02 | 800 | Germany | Control | 24 | GA | 0.0067 | 1 | 60 | 347,8408203 |
| 0005882_02 | 800 | Germany | Control | 24 | GA | 0.0067 | 1 | 14 | 112,9023438 |
| 0005882_02 | 800 | Germany | Control | 24 | GA | 0.0067 | 2 | 60 | 363,565918 |
| 0005882_02 | 800 | Germany | Control | 24 | GA | 0.0067 | 2 | 14 | 71,95947266 |
| 0005882_02 | 800 | Germany | Control | 25 | GA | 0.0067 | 1 | 60 | 431,6640625 |
| 0005882_02 | 800 | Germany | Control | 25 | GA | 0.0067 | 1 | 14 | 184,6166992 |
| 0005882_02 | 800 | Germany | Control | 25 | GA | 0.0067 | 2 | 60 | 441,3354492 |
| 0005882_02 | 800 | Germany | Control | 25 | GA | 0.0067 | 2 | 14 | 155,324707 |
| 0005882_02 | 800 | Germany | Control | 26 | GA | 0.0067 | 1 | 60 | 640,9775391 |
| 0005882_02 | 800 | Germany | Control | 26 | GA | 0.0067 | 1 | 14 | 112,7749023 |
| 0005882_02 | 800 | Germany | Control | 26 | GA | 0.0067 | 2 | 60 | 537,3696289 |
| 0005882_02 | 800 | Germany | Control | 26 | GA | 0.0067 | 2 | 14 | 103,5341797 |
| 0005900_01 | 800 | Germany | Control | 27 | GA | 0.0067 | 1 | 60 | 576,4121094 |
| 0005900_01 | 800 | Germany | Control | 27 | GA | 0.0067 | 1 | 14 | 228,0839844 |
| 0005900_01 | 800 | Germany | Control | 27 | GA | 0.0067 | 2 | 60 | 810,8022461 |
| 0005900_01 | 800 | Germany | Control | 27 | GA | 0.0067 | 2 | 14 | 221,0253906 |
| 0005900_01 | 800 | Germany | Control | 28 | GA | 0.0067 | 1 | 60 | 770,503418 |
| 0005900_01 | 800 | Germany | Control | 28 | GA | 0.0067 | 1 | 14 | 388,0322266 |
| 0005900_01 | 800 | Germany | Control | 28 | GA | 0.0067 | 2 | 60 | 822,387207 |
| 0005900_01 | 800 | Germany | Control | 28 | GA | 0.0067 | 2 | 14 | 397,6142578 |
| 0005900_01 | 800 | Germany | Control | 29 | GA | 0.0067 | 1 | 60 | 1351,291504 |
| 0005900_01 | 800 | Germany | Control | 29 | GA | 0.0067 | 1 | 14 | 202,7231445 |
| 0005900_01 | 800 | Germany | Control | 29 | GA | 0.0067 | 2 | 60 | 1400,081055 |
| 0005900_01 | 800 | Germany | Control | 29 | GA | 0.0067 | 2 | 14 | 197,7998047 |
| 0005900_01 | 800 | Germany | Control | 30 | GA | 0.0067 | 1 | 60 | 1454,668457 |
| 0005900_01 | 800 | Germany | Control | 30 | GA | 0.0067 | 1 | 14 | 169,4672852 |
| 0005900_01 | 800 | Germany | Control | 30 | GA | 0.0067 | 2 | 60 | 1347,973145 |
| 0005900_01 | 800 | Germany | Control | 30 | GA | 0.0067 | 2 | 14 | 145,8051758 |
| 0005900_01 | 800 | Germany | Control | 31 | GA | 0.0067 | 1 | 60 | 1175,161621 |
| 0005900_01 | 800 | Germany | Control | 31 | GA | 0.0067 | 1 | 14 | 132,5419922 |
| 0005900_01 | 800 | Germany | Control | 31 | GA | 0.0067 | 2 | 60 | 1137,947266 |
| 0005900_01 | 800 | Germany | Control | 31 | GA | 0.0067 | 2 | 14 | 116,8115234 |
| 0005900_01 | 800 | Germany | Control | 32 | GA | 0.0067 | 1 | 60 | 1087,254883 |
| 0005900_01 | 800 | Germany | Control | 32 | GA | 0.0067 | 1 | 14 | 329,8974609 |
| 0005900_01 | 800 | Germany | Control | 32 | GA | 0.0067 | 2 | 60 | 1102,499023 |
| 0005900_01 | 800 | Germany | Control | 32 | GA | 0.0067 | 2 | 14 | 337,3242188 |
| 0005900_01 | 800 | Germany | Control | 33 | GA | 0.0067 | 1 | 60 | 1521,009277 |
| 0005900_01 | 800 | Germany | Control | 33 | GA | 0.0067 | 1 | 14 | 375,206543 |
| 0005900_01 | 800 | Germany | Control | 33 | GA | 0.0067 | 2 | 60 | 1751,569336 |
| 0005900_01 | 800 | Germany | Control | 33 | GA | 0.0067 | 2 | 14 | 305,6787109 |
| 0005900_01 | 800 | Germany | Control | 34 | GA | 0.0067 | 1 | 60 | 1837,036621 |
| 0005900_01 | 800 | Germany | Control | 34 | GA | 0.0067 | 1 | 14 | 186,4086914 |
| 0005900_01 | 800 | Germany | Control | 34 | GA | 0.0067 | 2 | 60 | 1690,720703 |
| 0005900_01 | 800 | Germany | Control | 34 | GA | 0.0067 | 2 | 14 | 160,3662109 |
| 0006334_02 | 800 | Germany | Control | 35 | GA | 0.0067 | 1 | 60 | 513,4008789 |
| 0006334_02 | 800 | Germany | Control | 35 | GA | 0.0067 | 1 | 14 | 103,7871094 |
| 0006334_02 | 800 | Germany | Control | 35 | GA | 0.0067 | 2 | 60 | 807,8930664 |
| 0006334_02 | 800 | Germany | Control | 35 | GA | 0.0067 | 2 | 14 | 236,7636719 |
| 0006334_02 | 800 | Germany | Control | 36 | GA | 0.0067 | 1 | 60 | 1156,740234 |
| 0006334_02 | 800 | Germany | Control | 36 | GA | 0.0067 | 1 | 14 | 175,3676758 |
| 0006334_02 | 800 | Germany | Control | 36 | GA | 0.0067 | 2 | 60 | 1190,198242 |
| 0006334_02 | 800 | Germany | Control | 36 | GA | 0.0067 | 2 | 14 | 165,7070313 |
| 0006334_02 | 800 | Germany | Control | 37 | GA | 0.0067 | 1 | 60 | 1058,952637 |
| 0006334_02 | 800 | Germany | Control | 37 | GA | 0.0067 | 1 | 14 | 62,91015625 |
| 0006334_02 | 800 | Germany | Control | 37 | GA | 0.0067 | 2 | 60 | 1051,195801 |
| 0006334_02 | 800 | Germany | Control | 37 | GA | 0.0067 | 2 | 14 | 40,76025391 |
| 0006334_02 | 800 | Germany | Control | 38 | GA | 0.0067 | 1 | 60 | 855,8925781 |
| 0006334_02 | 800 | Germany | Control | 38 | GA | 0.0067 | 1 | 14 | 74,57226563 |
| 0006334_02 | 800 | Germany | Control | 38 | GA | 0.0067 | 2 | 60 | 901,7407227 |
| 0006334_02 | 800 | Germany | Control | 38 | GA | 0.0067 | 2 | 14 | 112,7705078 |
| 0006334_02 | 800 | Germany | Control | 39 | GA | 0.0067 | 1 | 60 | 687,8022461 |
| 0006334_02 | 800 | Germany | Control | 39 | GA | 0.0067 | 1 | 14 | 84,8828125 |
| 0006334_02 | 800 | Germany | Control | 39 | GA | 0.0067 | 2 | 60 | 762,59375 |
| 0006334_02 | 800 | Germany | Control | 39 | GA | 0.0067 | 2 | 14 | 48,96582031 |
| 0006334_02 | 800 | Germany | Control | 40 | GA | 0.0067 | 1 | 60 | 889,4404297 |
| 0006334_02 | 800 | Germany | Control | 40 | GA | 0.0067 | 1 | 14 | 143,6855469 |
| 0006334_02 | 800 | Germany | Control | 40 | GA | 0.0067 | 2 | 60 | 878,0537109 |
| 0006334_02 | 800 | Germany | Control | 40 | GA | 0.0067 | 2 | 14 | 74,16113281 |
| 0006334_02 | 800 | Germany | Control | 41 | GA | 0.0067 | 1 | 60 | 807,934082 |
| 0006334_02 | 800 | Germany | Control | 41 | GA | 0.0067 | 1 | 14 | 106,2050781 |
| 0006334_02 | 800 | Germany | Control | 41 | GA | 0.0067 | 2 | 60 | 656,9711914 |
| 0006334_02 | 800 | Germany | Control | 41 | GA | 0.0067 | 2 | 14 | 71,59570313 |
| 0006334_02 | 800 | Germany | Control | 42 | GA | 0.0067 | 1 | 60 | 1219,724121 |
| 0006334_02 | 800 | Germany | Control | 42 | GA | 0.0067 | 1 | 14 | 126,9306641 |
| 0006334_02 | 800 | Germany | Control | 42 | GA | 0.0067 | 2 | 60 | 977,5849609 |
| 0006334_02 | 800 | Germany | Control | 42 | GA | 0.0067 | 2 | 14 | 132,7880859 |
| 0006334_02 | 800 | Germany | Control | 43 | GA | 0.0067 | 1 | 60 | 629,5444336 |
| 0006334_02 | 800 | Germany | Control | 43 | GA | 0.0067 | 1 | 14 | 36,56542969 |
| 0006334_02 | 800 | Germany | Control | 43 | GA | 0.0067 | 2 | 60 | 552,7260742 |
| 0006334_02 | 800 | Germany | Control | 43 | GA | 0.0067 | 2 | 14 | 100,8125 |
| 0006334_02 | 800 | Germany | Control | 44 | GA | 0.0067 | 1 | 60 | 927,0092773 |
| 0006334_02 | 800 | Germany | Control | 44 | GA | 0.0067 | 1 | 14 | 130,7016602 |
| 0006334_02 | 800 | Germany | Control | 44 | GA | 0.0067 | 2 | 60 | 863,0439453 |
| 0006334_02 | 800 | Germany | Control | 44 | GA | 0.0067 | 2 | 14 | 101,2470703 |
| 0006334_02 | 800 | Germany | Control | 45 | GA | 0.0067 | 1 | 60 | 784,4492188 |
| 0006334_02 | 800 | Germany | Control | 45 | GA | 0.0067 | 1 | 14 | 67,07470703 |
| 0006334_02 | 800 | Germany | Control | 45 | GA | 0.0067 | 2 | 60 | 738,5605469 |
| 0006334_02 | 800 | Germany | Control | 45 | GA | 0.0067 | 2 | 14 | 150,1074219 |
| 0006334_02 | 800 | Germany | Control | 46 | GA | 0.0067 | 1 | 60 | 706,3149414 |
| 0006334_02 | 800 | Germany | Control | 46 | GA | 0.0067 | 1 | 14 | 87,50439453 |
| 0006334_02 | 800 | Germany | Control | 46 | GA | 0.0067 | 2 | 60 | 442,949707 |
| 0006334_02 | 800 | Germany | Control | 46 | GA | 0.0067 | 2 | 14 | 143,5473633 |
| 0006334_02 | 800 | Germany | Control | 47 | GA | 0.0067 | 1 | 60 | 589,4746094 |
| 0006334_02 | 800 | Germany | Control | 47 | GA | 0.0067 | 1 | 14 | 414,1455078 |
| 0006334_02 | 800 | Germany | Control | 47 | GA | 0.0067 | 2 | 60 | 638,0961914 |
| 0006334_02 | 800 | Germany | Control | 47 | GA | 0.0067 | 2 | 14 | 244,015625 |
| 0006334_02 | 800 | Germany | Control | 48 | GA | 0.0067 | 1 | 60 | 893,7724609 |
| 0006334_02 | 800 | Germany | Control | 48 | GA | 0.0067 | 1 | 14 | 398,9345703 |
| 0006334_02 | 800 | Germany | Control | 48 | GA | 0.0067 | 2 | 60 | 1038,928711 |
| 0006334_02 | 800 | Germany | Control | 48 | GA | 0.0067 | 2 | 14 | 614,6049805 |
| 0006334_02 | 800 | Germany | Control | 49 | GA | 0.0067 | 1 | 60 | 498,7104492 |
| 0006334_02 | 800 | Germany | Control | 49 | GA | 0.0067 | 1 | 14 | 132,9819336 |
| 0006334_02 | 800 | Germany | Control | 49 | GA | 0.0067 | 2 | 60 | 574,5571289 |
| 0006334_02 | 800 | Germany | Control | 49 | GA | 0.0067 | 2 | 14 | 64,66357422 |
| 0006334_02 | 800 | Germany | Control | 50 | GA | 0.0067 | 1 | 60 | 755,1049805 |
| 0006334_02 | 800 | Germany | Control | 50 | GA | 0.0067 | 1 | 14 | 87,10351563 |
| 0006334_02 | 800 | Germany | Control | 50 | GA | 0.0067 | 2 | 60 | 717,015625 |
| 0006334_02 | 800 | Germany | Control | 50 | GA | 0.0067 | 2 | 14 | 121,2353516 |
| 0006334_02 | 800 | Germany | Control | 51 | GA | 0.0067 | 1 | 60 | 621,6196289 |
| 0006334_02 | 800 | Germany | Control | 51 | GA | 0.0067 | 1 | 14 | 111,8886719 |
| 0006334_02 | 800 | Germany | Control | 51 | GA | 0.0067 | 2 | 60 | 576,9267578 |
| 0006334_02 | 800 | Germany | Control | 51 | GA | 0.0067 | 2 | 14 | 84,89501953 |
| 0006371_02 | 800 | Germany | Control | 52 | GA | 0.0067 | 1 | 60 | 673,8652344 |
| 0006371_02 | 800 | Germany | Control | 52 | GA | 0.0067 | 1 | 14 | 231,0087891 |
| 0006371_02 | 800 | Germany | Control | 52 | GA | 0.0067 | 2 | 60 | 725,0390625 |
| 0006371_02 | 800 | Germany | Control | 52 | GA | 0.0067 | 2 | 14 | 182,2431641 |
| 0006371_02 | 800 | Germany | Control | 53 | GA | 0.0067 | 1 | 60 | 881,5166016 |
| 0006371_02 | 800 | Germany | Control | 53 | GA | 0.0067 | 1 | 14 | 204,6962891 |
| 0006371_02 | 800 | Germany | Control | 53 | GA | 0.0067 | 2 | 60 | 730,4082031 |
| 0006371_02 | 800 | Germany | Control | 53 | GA | 0.0067 | 2 | 14 | 264,8808594 |
| 0006371_02 | 800 | Germany | Control | 54 | GA | 0.0067 | 1 | 60 | 428,7558594 |
| 0006371_02 | 800 | Germany | Control | 54 | GA | 0.0067 | 1 | 14 | 501,0244141 |
| 0006371_02 | 800 | Germany | Control | 54 | GA | 0.0067 | 2 | 60 | 823,7568359 |
| 0006371_02 | 800 | Germany | Control | 54 | GA | 0.0067 | 2 | 14 | 589,9960938 |
| 0006371_02 | 800 | Germany | Control | 55 | GA | 0.0067 | 1 | 60 | 908,8984375 |
| 0006371_02 | 800 | Germany | Control | 55 | GA | 0.0067 | 1 | 14 | 208,8291016 |
| 0006371_02 | 800 | Germany | Control | 55 | GA | 0.0067 | 2 | 60 | 593,2744141 |
| 0006371_02 | 800 | Germany | Control | 55 | GA | 0.0067 | 2 | 14 | 232,1904297 |
| 0006371_02 | 800 | Germany | Control | 56 | GA | 0.0067 | 1 | 60 | 545,2255859 |
| 0006371_02 | 800 | Germany | Control | 56 | GA | 0.0067 | 1 | 14 | 205,0058594 |
| 0006371_02 | 800 | Germany | Control | 56 | GA | 0.0067 | 2 | 60 | 747,3847656 |
| 0006371_02 | 800 | Germany | Control | 56 | GA | 0.0067 | 2 | 14 | 165,1396484 |
| 0006371_02 | 800 | Germany | Control | 57 | GA | 0.0067 | 1 | 60 | 416,8808594 |
| 0006371_02 | 800 | Germany | Control | 57 | GA | 0.0067 | 1 | 14 | 326,3398438 |
| 0006371_02 | 800 | Germany | Control | 57 | GA | 0.0067 | 2 | 60 | 320,2490234 |
| 0006371_02 | 800 | Germany | Control | 57 | GA | 0.0067 | 2 | 14 | 358,7568359 |
| 0006371_02 | 800 | Germany | Control | 58 | GA | 0.0067 | 1 | 60 | 447,0556641 |
| 0006371_02 | 800 | Germany | Control | 58 | GA | 0.0067 | 1 | 14 | 164,8457031 |
| 0006371_02 | 800 | Germany | Control | 58 | GA | 0.0067 | 2 | 60 | 540,3212891 |
| 0006371_02 | 800 | Germany | Control | 58 | GA | 0.0067 | 2 | 14 | 247,9960938 |
| 0006371_02 | 800 | Germany | Control | 59 | GA | 0.0067 | 1 | 60 | 591,7060547 |
| 0006371_02 | 800 | Germany | Control | 59 | GA | 0.0067 | 1 | 14 | 232,2475586 |
| 0006371_02 | 800 | Germany | Control | 59 | GA | 0.0067 | 2 | 60 | 494,5292969 |
| 0006371_02 | 800 | Germany | Control | 59 | GA | 0.0067 | 2 | 14 | 285,0810547 |
| 0006371_02 | 800 | Germany | Control | 60 | GA | 0.0067 | 1 | 60 | 657,9472656 |
| 0006371_02 | 800 | Germany | Control | 60 | GA | 0.0067 | 1 | 14 | 222,1259766 |
| 0006371_02 | 800 | Germany | Control | 60 | GA | 0.0067 | 2 | 60 | 734,9306641 |
| 0006371_02 | 800 | Germany | Control | 60 | GA | 0.0067 | 2 | 14 | 175,6035156 |
| 0006371_02 | 800 | Germany | Control | 61 | GA | 0.0067 | 1 | 60 | 974,3876953 |
| 0006371_02 | 800 | Germany | Control | 61 | GA | 0.0067 | 1 | 14 | 252,519043 |
| 0006371_02 | 800 | Germany | Control | 61 | GA | 0.0067 | 2 | 60 | 871,7583008 |
| 0006371_02 | 800 | Germany | Control | 61 | GA | 0.0067 | 2 | 14 | 288,3198242 |
| 0006371_02 | 800 | Germany | Control | 62 | GA | 0.0067 | 1 | 60 | 1313,043945 |
| 0006371_02 | 800 | Germany | Control | 62 | GA | 0.0067 | 1 | 14 | 391,4111328 |
| 0006371_02 | 800 | Germany | Control | 62 | GA | 0.0067 | 2 | 60 | 1266,536133 |
| 0006371_02 | 800 | Germany | Control | 62 | GA | 0.0067 | 2 | 14 | 513,6044922 |
| 0006371_02 | 800 | Germany | Control | 63 | GA | 0.0067 | 1 | 60 | 494,4706116 |
| 0006371_02 | 800 | Germany | Control | 63 | GA | 0.0067 | 1 | 14 | 351,7109375 |
| 0006371_02 | 800 | Germany | Control | 63 | GA | 0.0067 | 2 | 60 | 325,5180664 |
| 0006371_02 | 800 | Germany | Control | 63 | GA | 0.0067 | 2 | 14 | 510,7651367 |
| 0006371_02 | 800 | Germany | Control | 64 | GA | 0.0067 | 1 | 60 | 904,5444336 |
| 0006371_02 | 800 | Germany | Control | 64 | GA | 0.0067 | 1 | 14 | 327,4702148 |
| 0006371_02 | 800 | Germany | Control | 64 | GA | 0.0067 | 2 | 60 | 1000,943359 |
| 0006371_02 | 800 | Germany | Control | 64 | GA | 0.0067 | 2 | 14 | 321,9238281 |
| 0006371_02 | 800 | Germany | Control | 65 | GA | 0.0067 | 1 | 60 | 1378,400879 |
| 0006371_02 | 800 | Germany | Control | 65 | GA | 0.0067 | 1 | 14 | 625,5991211 |
| 0006371_02 | 800 | Germany | Control | 65 | GA | 0.0067 | 2 | 60 | 1569,319824 |
| 0006371_02 | 800 | Germany | Control | 65 | GA | 0.0067 | 2 | 14 | 605,4238281 |
| 0006371_02 | 800 | Germany | Control | 66 | GA | 0.0067 | 1 | 60 | 933,7177734 |
| 0006371_02 | 800 | Germany | Control | 66 | GA | 0.0067 | 1 | 14 | 396,2285156 |
| 0006371_02 | 800 | Germany | Control | 66 | GA | 0.0067 | 2 | 60 | 891,6508789 |
| 0006371_02 | 800 | Germany | Control | 66 | GA | 0.0067 | 2 | 14 | 210,6777344 |
| 0006371_02 | 800 | Germany | Control | 67 | GA | 0.0067 | 1 | 60 | 834,4951172 |
| 0006371_02 | 800 | Germany | Control | 67 | GA | 0.0067 | 1 | 14 | 141,605957 |
| 0006371_02 | 800 | Germany | Control | 67 | GA | 0.0067 | 2 | 60 | 328,394043 |
| 0006371_02 | 800 | Germany | Control | 67 | GA | 0.0067 | 2 | 14 | 173,6967773 |
| 0006371_02 | 800 | Germany | Control | 68 | GA | 0.0067 | 1 | 60 | 215,6445313 |
| 0006371_02 | 800 | Germany | Control | 68 | GA | 0.0067 | 1 | 14 | 21,13720703 |
| 0006371_02 | 800 | Germany | Control | 68 | GA | 0.0067 | 2 | 60 | 626,2587891 |
| 0006371_02 | 800 | Germany | Control | 68 | GA | 0.0067 | 2 | 14 | 118,53125 |
| 0006371_02 | 800 | Germany | Control | 69 | GA | 0.0067 | 1 | 60 | 905,546875 |
| 0006371_02 | 800 | Germany | Control | 69 | GA | 0.0067 | 1 | 14 | 286,5166016 |
| 0006371_02 | 800 | Germany | Control | 69 | GA | 0.0067 | 2 | 60 | 819,2177734 |
| 0006371_02 | 800 | Germany | Control | 69 | GA | 0.0067 | 2 | 14 | 212,4697266 |
| 0006371_02 | 800 | Germany | Control | 70 | GA | 0.0067 | 1 | 60 | 588,8408203 |
| 0006371_02 | 800 | Germany | Control | 70 | GA | 0.0067 | 1 | 14 | 424,7197266 |
| 0006371_02 | 800 | Germany | Control | 70 | GA | 0.0067 | 2 | 60 | 557,4355469 |
| 0006371_02 | 800 | Germany | Control | 70 | GA | 0.0067 | 2 | 14 | 273,4541016 |
| 0006371_02 | 800 | Germany | Control | 71 | GA | 0.0067 | 1 | 60 | 1397,398438 |
| 0006371_02 | 800 | Germany | Control | 71 | GA | 0.0067 | 1 | 14 | 348,1269531 |
| 0006371_02 | 800 | Germany | Control | 71 | GA | 0.0067 | 2 | 60 | 1127,962402 |
| 0006371_02 | 800 | Germany | Control | 71 | GA | 0.0067 | 2 | 14 | 303,9746094 |
| 0006371_02 | 800 | Germany | Control | 72 | GA | 0.0067 | 1 | 60 | 1130,65332 |
| 0006371_02 | 800 | Germany | Control | 72 | GA | 0.0067 | 1 | 14 | 65,00097656 |
| 0006371_02 | 800 | Germany | Control | 72 | GA | 0.0067 | 2 | 60 | 990,8691406 |
| 0006371_02 | 800 | Germany | Control | 72 | GA | 0.0067 | 2 | 14 | 126,9892578 |
| 0006296_01 | 800 | Germany | Control | 73 | GA | 0.0067 | 1 | 60 | 1776,585449 |
| 0006296_01 | 800 | Germany | Control | 73 | GA | 0.0067 | 1 | 14 | 349,2041016 |
| 0006296_01 | 800 | Germany | Control | 73 | GA | 0.0067 | 2 | 60 | 1446,830078 |
| 0006296_01 | 800 | Germany | Control | 73 | GA | 0.0067 | 2 | 14 | 609,9150391 |
| 0006296_01 | 800 | Germany | Control | 74 | GA | 0.0067 | 1 | 60 | 950,2490234 |
| 0006296_01 | 800 | Germany | Control | 74 | GA | 0.0067 | 1 | 14 | 292,7402344 |
| 0006296_01 | 800 | Germany | Control | 74 | GA | 0.0067 | 2 | 60 | 1257,619141 |
| 0006296_01 | 800 | Germany | Control | 74 | GA | 0.0067 | 2 | 14 | 217,8320313 |
| 0006296_01 | 800 | Germany | Control | 75 | GA | 0.0067 | 1 | 60 | 1848,370117 |
| 0006296_01 | 800 | Germany | Control | 75 | GA | 0.0067 | 1 | 14 | 378,0839844 |
| 0006296_01 | 800 | Germany | Control | 75 | GA | 0.0067 | 2 | 60 | 1829,294922 |
| 0006296_01 | 800 | Germany | Control | 75 | GA | 0.0067 | 2 | 14 | 381,8359375 |
| 0006371_02 | 800 | Germany | Control | 76 | GA | 0.0067 | 1 | 60 | 801,6572266 |
| 0006371_02 | 800 | Germany | Control | 76 | GA | 0.0067 | 1 | 14 | 122,5205078 |
| 0006371_02 | 800 | Germany | Control | 76 | GA | 0.0067 | 2 | 60 | 727,8457031 |
| 0006371_02 | 800 | Germany | Control | 76 | GA | 0.0067 | 2 | 14 | 211,1015625 |
| 0006371_02 | 800 | Germany | Control | 77 | GA | 0.0067 | 1 | 60 | 592,1289063 |
| 0006371_02 | 800 | Germany | Control | 77 | GA | 0.0067 | 1 | 14 | 251,6621094 |
| 0006371_02 | 800 | Germany | Control | 77 | GA | 0.0067 | 2 | 60 | 327,1962891 |
| 0006371_02 | 800 | Germany | Control | 77 | GA | 0.0067 | 2 | 14 | 124,2890625 |
| 0006371_02 | 800 | Germany | Control | 78 | GA | 0.0067 | 1 | 60 | 997,2392578 |
| 0006371_02 | 800 | Germany | Control | 78 | GA | 0.0067 | 1 | 14 | 198,7832031 |
| 0006371_02 | 800 | Germany | Control | 78 | GA | 0.0067 | 2 | 60 | 647,6015625 |
| 0006371_02 | 800 | Germany | Control | 78 | GA | 0.0067 | 2 | 14 | 197,0478516 |
| 0006371_02 | 800 | Germany | Control | 79 | GA | 0.0067 | 1 | 60 | 391,8564453 |
| 0006371_02 | 800 | Germany | Control | 79 | GA | 0.0067 | 1 | 14 | 328,7695313 |
| 0006371_02 | 800 | Germany | Control | 79 | GA | 0.0067 | 2 | 60 | 570,5986328 |
| 0006371_02 | 800 | Germany | Control | 79 | GA | 0.0067 | 2 | 14 | 408,7919922 |
| 0006371_02 | 800 | Germany | Control | 80 | GA | 0.0067 | 1 | 60 | 695,0927734 |
| 0006371_02 | 800 | Germany | Control | 80 | GA | 0.0067 | 1 | 14 | 314,6411133 |
| 0006371_02 | 800 | Germany | Control | 80 | GA | 0.0067 | 2 | 60 | 697,5795898 |
| 0006371_02 | 800 | Germany | Control | 80 | GA | 0.0067 | 2 | 14 | 199,5263672 |
| 0006334_02 | 800 | Germany | Control | 81 | GA | 0.0067 | 1 | 60 | 1062,643066 |
| 0006334_02 | 800 | Germany | Control | 81 | GA | 0.0067 | 1 | 14 | 209,5859375 |
| 0006334_02 | 800 | Germany | Control | 81 | GA | 0.0067 | 2 | 60 | 876,8676758 |
| 0006334_02 | 800 | Germany | Control | 81 | GA | 0.0067 | 2 | 14 | 212,4106445 |
| 0006334_02 | 800 | Germany | Control | 82 | GA | 0.0067 | 1 | 60 | 751,1796875 |
| 0006334_02 | 800 | Germany | Control | 82 | GA | 0.0067 | 1 | 14 | 350,1728516 |
| 0006334_02 | 800 | Germany | Control | 82 | GA | 0.0067 | 2 | 60 | 807,8837891 |
| 0006334_02 | 800 | Germany | Control | 82 | GA | 0.0067 | 2 | 14 | 392,1542969 |
| 0006334_02 | 800 | Germany | Control | 83 | GA | 0.0067 | 1 | 60 | 559,0532227 |
| 0006334_02 | 800 | Germany | Control | 83 | GA | 0.0067 | 1 | 14 | 162,1728516 |
| 0006334_02 | 800 | Germany | Control | 83 | GA | 0.0067 | 2 | 60 | 476,7485352 |
| 0006334_02 | 800 | Germany | Control | 83 | GA | 0.0067 | 2 | 14 | 222,4477539 |

DSG=Disuccinimidyl glutarate, GA=glutaraldehyde; f.c.=final concentration (DSG in mM, GA in %), kDA=kilo Dalton, sPD=sporadic Parkinsoon`s disease
