# Supplementary material for: Aggregation-resistant alpha-synuclein tetramers are reduced in the blood of Parkinson’s patients
Source: EMBO Mol Med. 2024 Jun 5;16(7):10. doi: 10.1038/s44321-024-00083-5 (PMC11250827; doi:10.1038/s44321-024-00083-5)
Supplement: Supplementary file 7 — Expanded View Figures [file 44321_2024_83_MOESM7_ESM.pdf]

Expanded View Figures

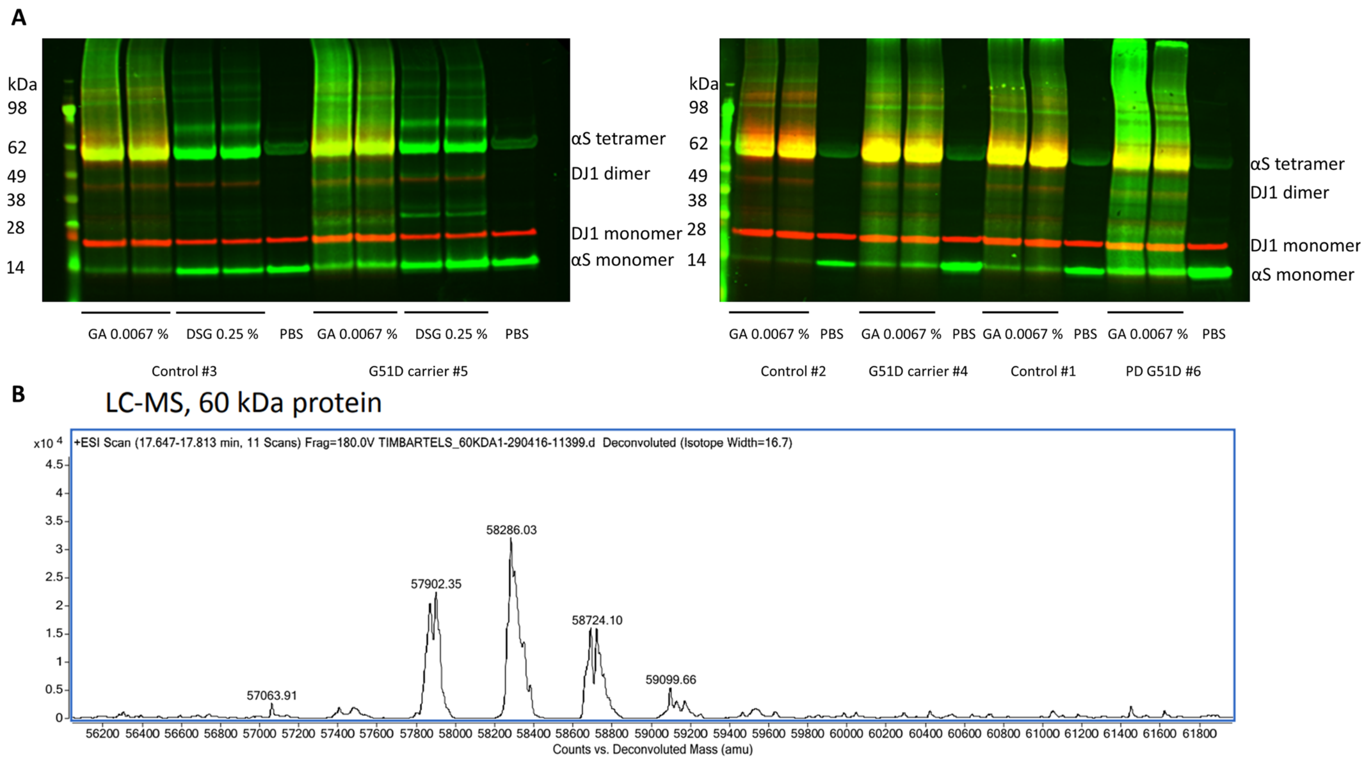

Figure EV1. Representative Western blot and Mass spectrometry analysis.

(**A**) Western blot analysis of G51D carriers and one PD G51D patient blood lysate compared to controls. Representative pictures of Western blot analyses from controls, G51D carriers and one PD patient with a G51D mutation using the cross-linker GA and DSG. All samples have been analyzed in technical duplicates. Information on clinical characteristics is provided in Table 1. Samples for Fig. EV1 (depleting hemoglobin, cross-linking, gels, blots) were processed in parallel on different blots due to the samples size. No loading controls were run on the western blot as the full volume of each processed sample containing 20 µg of total protein was loaded into each gel pocket. (**B**) Mass spectrometry traces of immunoprecipitated blood-derived α-synuclein. The graph displays the expected mass for isolated tetrameric α-synuclein: 58,286 kDa. In comparison, the expected mass for the DSG cross-linker (unconjugated) would be 326 kDa. Source data are available online for this figure.

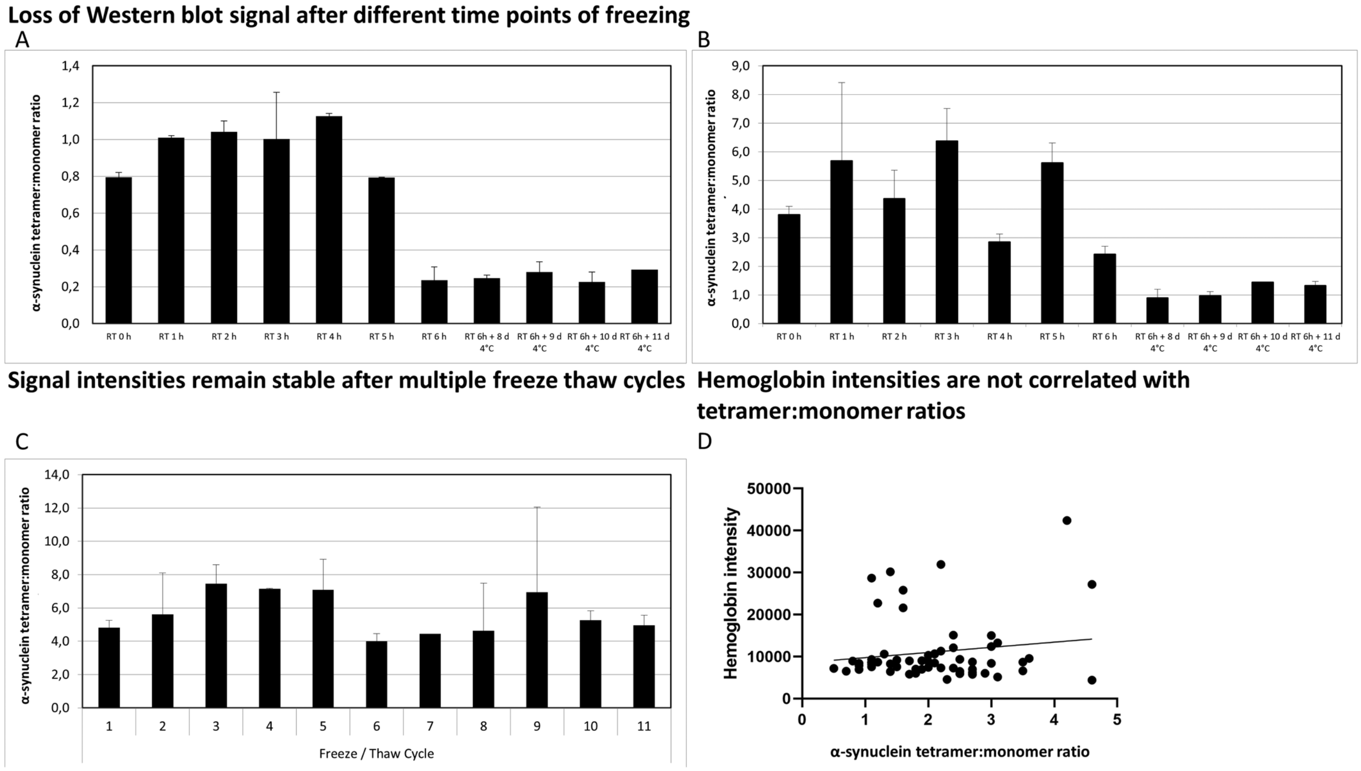

Figure EV2. Validation of the cross-linking protocol.

The procedure is described in the Methods section. (**A**) Quantification of the Western blot of cross-linked (DSG) human whole blood after Hemoglobin depletion (ratio Blood:HemogloBind 1:1, $n = 1$, control sample). Blood samples were left at room temperature for different time points (0–6 h) or placed at 4 °C for 8–11 days after the samples have been kept at 6 h RT. Samples were analyzed in two technical replicates. Due to degradation processes, the signal/ratio drops after 6 h at RT and subsequent cooling at 4 °C. Data is displayed as mean ± s.d. (**B**) Quantification of the Western blot of cross-linked (DSG) human whole blood after Hemoglobin depletion (ratio Blood:HemogloBind 1:4, $n = 1$, control sample). Blood samples were left at room temperature for different time points (0–6 h) or placed at 4 °C for 8–11 days after the samples have been kept at 6 h RT. Samples were analyzed in two technical replicates. Due to degradation processes, the signal/ratio drops after 6 h at RT and subsequent cooling at 4 °C. Data is displayed as mean ± s.d. (**C**) Signal intensities of the Western blot analysis remain stable after multiple freeze/thaw cycles (Blood:HemogloBind 1:1, $n = 1$, control sample). Samples were analyzed in two technical replicates. Data is displayed as mean ± s.d. (**D**) Hemoglobin intensities after removal of Hemoglobin were correlated (Pearson correlation) with α-synuclein tetramer:monomer ratios (Blood:HemogloBind 1:1, $r = 0.2$, $p = 0.2$, $n = 60$). RT room temperature, DSG Disuccinimidyl glutarate.

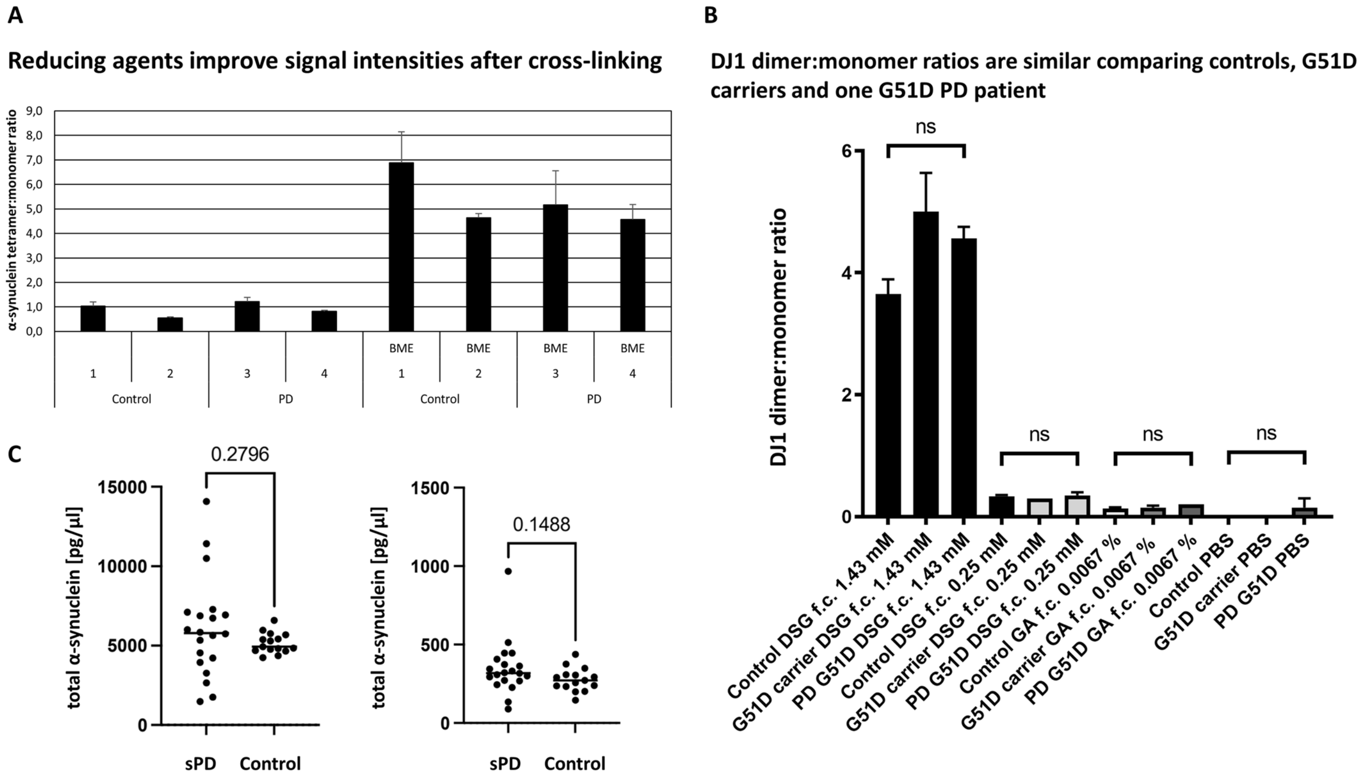

Figure EV3. Validation of the cross-linking protocol.

(**A**) Validation of the cross-linking protocol. The procedure is described in the Methods section. Quantification of the Western blot of cross-linked (DSG) human whole blood after Hemoglobin depletion (ratio Blood:HemogloBind 1:1, $n = 4$, 2 control samples, 2 PD samples). The signal is increased after reducing the sample at 70 °C using 4 × NuPage LDS sample buffer (Novex)/1:10 β-mercaptoethanol (Sigma). Samples were analyzed in two technical replicates. Data is displayed as mean ± s.d. BME = β-mercaptoethanol, PD = Parkinson's disease, DSG=Disuccinimidyl glutarate. (**B**) DJ1 dimer:monomer ratios are similar between controls, G51D carriers and PD G51D patients. The DJ1 protein serves as an internal control for the cross-linking procedure. All groups were compared using Mann–Whitney-U test. G51D carriers $n = 2$, G51D PD patient ($n = 1$), controls $n = 3$. Samples were analyzed in two technical replicates. Data is displayed as mean ± s.d. F.c. = final concentration, PD = Parkinson's disease, DSG = Disuccinimidyl glutarate, GA = glutaraldehyde. (**C**) Analysis of total α-synuclein blood levels. Left, total, blood-derived cytosolic α-synuclein levels do not differ between sPD patients ($n = 20$) and controls ($n = 15$, $p = 0.3$). Cytosolic, soluble α-synuclein was derived after mechanical cell lysis. Right, total, blood-derived membrane-associated α-synuclein levels do not differ between sPD patients ($n = 20$) and controls ($n = 15$, $p = 0.2$). Membrane-associated α-synuclein was derived after mechanical cell lysis from the 1% Triton-soluble fraction. All Samples were analyzed in two technical replicates. All groups were compared using Mann–Whitney-U test. Mean for each sample is displayed.

 © The Author(s)

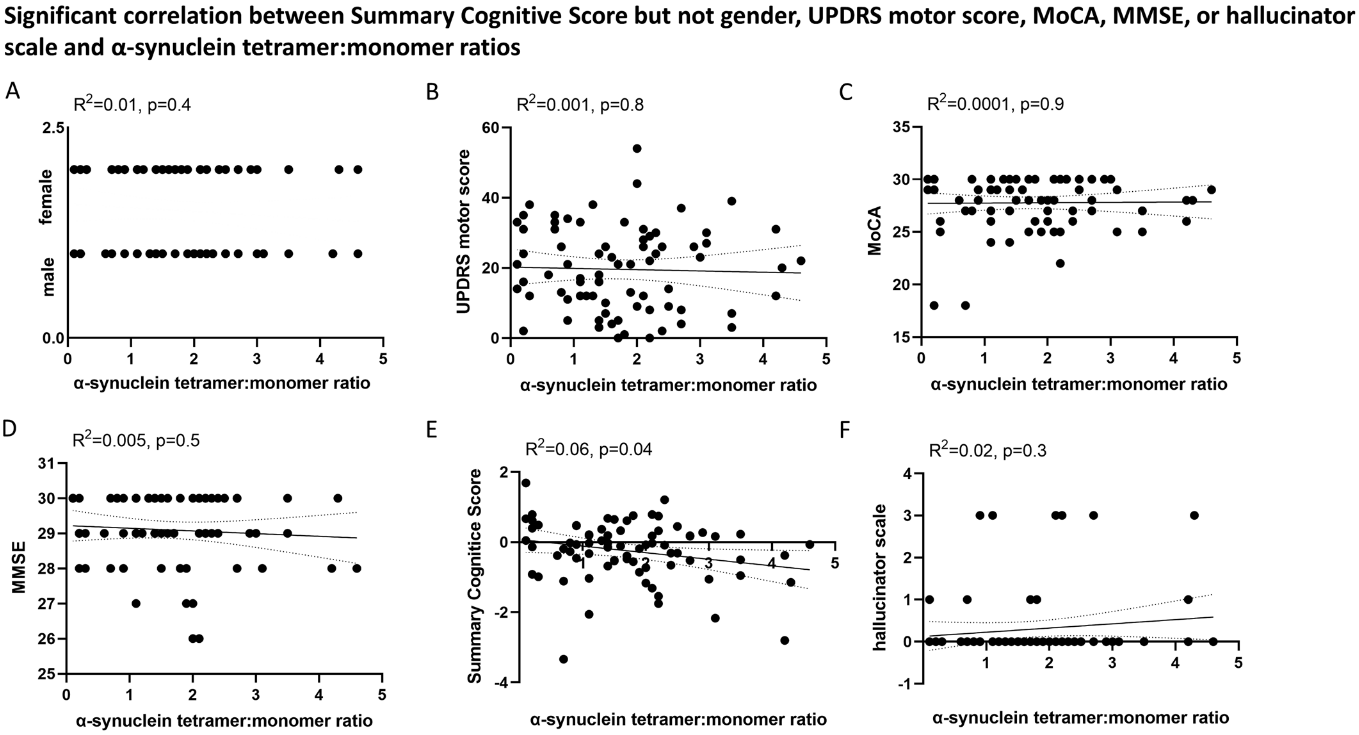

Figure EV4. Correlation analysis of α-synuclein tetramer:monomer ratios and clinical parameters.

Pearson correlation of cohort 1 shows Pearson's correlation analysis of α-synuclein tetramer:monomer ratios and (**A**) gender, (**B**) UPDRS motor score, (**C**) MoCA, (**D**) MMSE, (**E**) Summary Cognitive Score, (**F**) hallucinator scale.

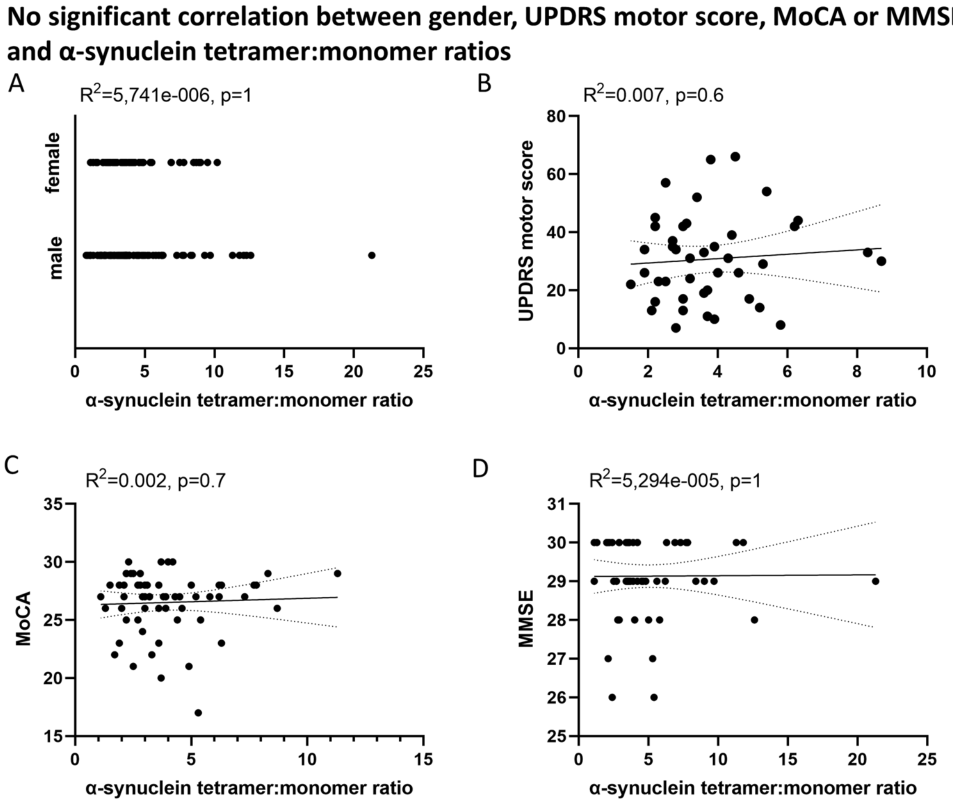

Figure EV5. Correlation analysis of α-synuclein tetramer:monomer ratios and clinical parameters.

Pearson correlation of cohort 2 shows no significant correlation of α-synuclein tetramer:monomer ratios and (**A**) gender, (**B**) UPDRS motor score, (**C**) MoCA, (**D**) MMSE.

 © The Author(s)
